# Sites of transcription initiation drive mRNA isoform selection

**DOI:** 10.1016/j.cell.2023.04.012

**Published:** 2023-05-25

**Authors:** Carlos Alfonso-Gonzalez, Ivano Legnini, Sarah Holec, Laura Arrigoni, Hasan Can Ozbulut, Fernando Mateos, David Koppstein, Agnieszka Rybak-Wolf, Ulrike Bönisch, Nikolaus Rajewsky, Valérie Hilgers

**Affiliations:** 1 Max-Planck-Institute of Immunobiology and Epigenetics, 79108 Freiburg, Germany; 2 Faculty of Biology, Albert Ludwig University, 79104 Freiburg, Germany; 3 International Max Planck Research School for Molecular and Cellular Biology (IMPRS-MCB), 79108 Freiburg, Germany; 4 Laboratory for Systems Biology of Gene Regulatory Elements, Berlin Institute for Medical Systems Biology, Max Delbrück Center for Molecular Medicine in the Helmholtz Association, 10115 Berlin, Germany; 5 Organoid Platform, Berlin Institute for Medical Systems Biology, Max Delbrück Center for Molecular Medicine in the Helmholtz Association, 10115 Berlin, Germany; 6 Charité - Universitätsmedizin, Charitépl. 1, 10117 Berlin, Germany; 7 German Center for Cardiovascular Research (DZHK), Site Berlin, Berlin, Germany; 8 NeuroCure Cluster of Excellence, Berlin, Germany; 9 German Cancer Consortium (DKTK); 10 National Center for Tumor Diseases (NCT), Site Berlin, Berlin, Germany; 11 Signalling Research Centre CIBSS, University of Freiburg, Schänzlestraße 18, 79104 Freiburg, Germany

**Keywords:** transcription, mRNA isoform, 5ʹ-3ʹ coupling, transcription start site, alternative polyadenylation, long-read sequencing, *Drosophila*, human brain organoids, nervous system, p300/CBP

## Abstract

The generation of distinct messenger RNA isoforms through alternative RNA processing modulates the expression and function of genes, often in a cell-type-specific manner. Here, we assess the regulatory relationships between transcription initiation, alternative splicing, and 3′ end site selection. Applying long-read sequencing to accurately represent even the longest transcripts from end to end, we quantify mRNA isoforms in *Drosophila* tissues, including the transcriptionally complex nervous system. We find that in *Drosophila* heads, as well as in human cerebral organoids, 3′ end site choice is globally influenced by the site of transcription initiation (TSS). “Dominant promoters,” characterized by specific epigenetic signatures including p300/CBP binding, impose a transcriptional constraint to define splice and polyadenylation variants. *In vivo* deletion or overexpression of dominant promoters as well as p300/CBP loss disrupted the 3′ end expression landscape. Our study demonstrates the crucial impact of TSS choice on the regulation of transcript diversity and tissue identity.

## Highlights

• A quantification of *Drosophila* and human nervous system full-length mRNA isoforms
• 3′ end site selection is coupled to alternative TSS usage
• Dominant promoters drive alternative polyadenylation through p300/CBP
• Conserved 5′-3′ couplings regulate tissue-specific functions

## Graphical abstract

**Figure:**
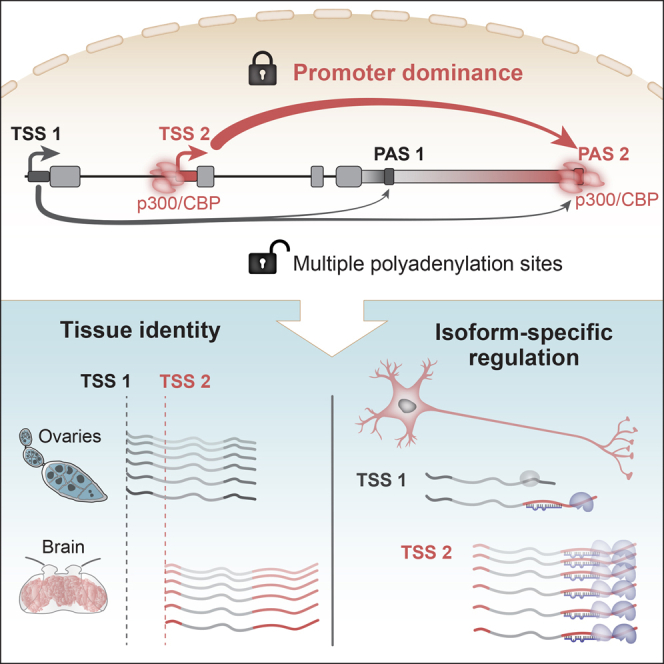

## Teaser

Where an mRNA transcript starts determines where it ends. Epigenetic marks characterize “dominant promoters” that constrain start and polyadenylation sites, driving tissue-specific preferential expression of transcript variants.

## Introduction

Variation at each step of pre-messenger RNA (mRNA) synthesis impacts the coding and non-coding content of the mature transcript. Alternative splicing (AS) and alternative polyadenylation (APA) generate mRNA isoforms that differ in their coding sequence (CDS) or the length of their 3′ untranslated region (3′ UTR), thereby contributing to proteome diversity and fine-tuning gene expression. Alternative 3′ UTRs, through distinct sequence and structure elements that dictate interactions of the transcript with microRNAs and RNA-binding proteins (RBPs), regulate the encoded protein’s abundance, localization, and integration into protein complexes.^1^ APA modulates protein function in a context-specific, gene-specific, or cell-type-specific manner and is critically involved in a variety of cellular processes; indeed, numerous human diseases including cancer and neurological disorders^2^^,^^3^ are associated with APA deregulation. 3′ UTR genetic variants contribute to a substantial number of phenotypic traits and disease heritability,^4^^,^^5^ making APA a possible actionable target for therapeutic intervention.

The tissue- or context-specific regulation of APA is mediated through the activity of effectors such as transcription factors or RBPs. For example, in animals from flies to humans, the neuron-specific ELAV/Hu proteins inhibit splice site and proximal polyadenylation (poly(A)) site usage to mediate the formation of neuronal 3′ UTRs.^6^ Depending on cellular context, transcription elongation and termination factors interact with the cleavage and polyadenylation (CPA) machinery to enhance or inhibit 3′ end processing.^7^^,^^8^^,^^9^^,^^10^ The gene-specific regulation of APA is less well understood. Alternative 3′ UTR formation in individual mRNAs was shown to depend on sequence elements located in promoters or enhancers.^11^^,^^12^ Several studies provide evidence of a physical connection between transcription start sites (TSSs) and poly(A) sites (PASs): RBPs pervasively associate with promoter regions, as does the CPA machinery.^13^^,^^14^^,^^15^^,^^16^ Moreover, DNA methylation and CTCF recruitment influence APA,^17^ and gene loops affect alternative 3′ end processing in yeast,^18^ indicating a possible role for chromatin looping in 3′ end site selection. Together, such observations suggest that transcription regulation at promoters may be functionally coupled with APA; however, whether TSSs globally influence the selection of PASs remains unknown.^19^

The main challenge in determining the regulatory links that mediate the choice of transcription initiation, splicing, and termination sites has been the ability to correlate different regions of a single transcript to one another—in particular, the 5′ end and the 3′ end of the same mRNA molecule, which typically lie several kilobases (kb) apart. Long-read sequencing (LRS) technologies now allow for full delineation of individual mRNA isoforms: in a single read, transcript coverage can be achieved from 5′ to 3′ end.^20^^,^^21^ LRS has been successfully used for the discovery of novel transcripts from repetitive regions, detection of novel splice variants, identification of interactions between alternative promoters and splicing of promoter-proximal exons, and for the identification of coupling events in feature pairs including TSSs, exons, and PASs.^22^^,^^23^^,^^24^^,^^25^^,^^26^^,^^27^^,^^28^ Short-read sequencing and LRS of nascent RNAs have shed light on intertwined co-transcriptional processes^29^^,^^30^ and demonstrated, for example, the influence of splicing dynamics on CPA efficiency,^31^^,^^32^ indicating a widespread interdependency between alternative transcription and RNA processing. However, so far, technologies have failed to resolve the link between 5′ ends and 3′ ends. Transcript isoform sequencing approaches that concurrently determine the start and end sites of individual RNA molecules, although well suited for determining transcript boundaries and their combinations,^33^ have not been employed to quantify couplings between 5′ and 3′ ends. Major limitations have indeed precluded the systematic analysis of the regulatory relationship between transcription initiation and termination. LRS read distributions typically peak at 1–2 kb in length, resulting in truncations, underrepresentation of long isoforms, and 5′ or 3′ sequencing biases.^22^^,^^34^ As a result, due to the incomplete representation of full-length mRNA isoforms, it has not been possible to quantify the contribution of different TSSs of the same gene to the expression of distinct 3′ ends.

Here, we analyze the co-occurrence of mRNA features at the isoform level in the *Drosophila* nervous system, which is characterized by a particularly diverse transcriptome. We used multiple LRS approaches and developed a framework to accurately assess and quantify mRNA isoform usage, including the definition of true PASs. Our data demonstrate coupling between transcript 5′ ends and 3′ ends. We identify “dominant” promoters that, characterized by a unique epigenetic signature, outcompete cognate promoters to drive the expression of alternative, usually more distal, 3′ ends. Promoter dominance is widespread in *Drosophila* brains and human cerebral organoids and constitutes a major mechanism to regulate 3′ end site choice during transcription to generate select 5′ UTR-3′ UTR combinations in mature mRNAs.

## Results

### A combined isoform assembly reflects the *Drosophila* transcriptome

**Figure 1:**
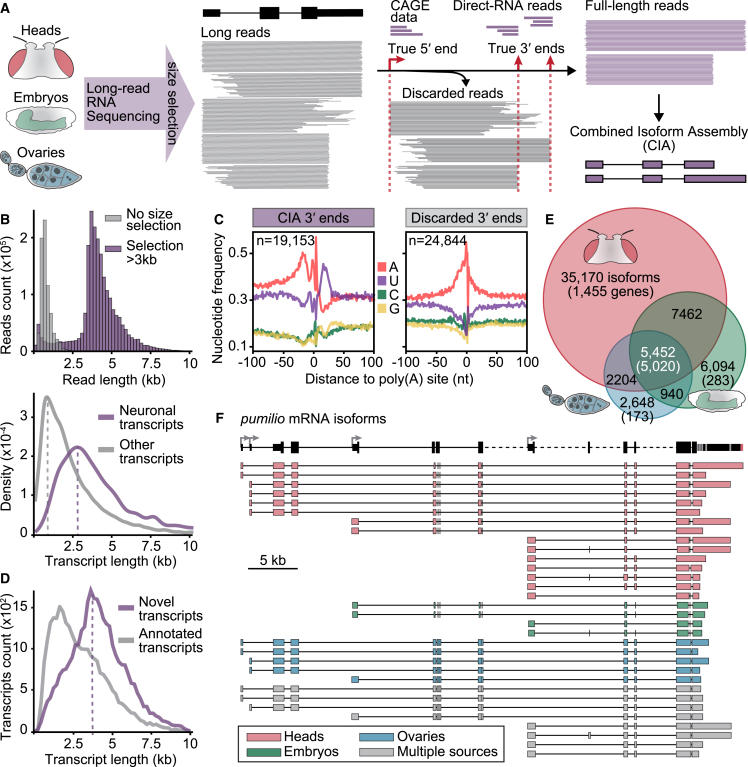
An accurate, comprehensive, full-length *Drosophila* transcriptome (A) Combined isoform assembly (CIA) experimental and computational workflow. Long-read sequencing was performed on three *Drosophila* tissues: adult heads, embryos at the developmental time points 14–16 h after egg laying (AEL) and 18–20 h AEL, and adult ovaries. Transcript size selection was performed to optimize recovery of neuronal transcripts. The final transcriptome assembly was built on full-length reads, i.e., those that spanned an entire mRNA transcript isoform from experimentally validated TSS (true 5′ end) to experimentally validated 3′ end (true 3′ end). Individual reads are represented as straight lines spanning different regions of the gene. (B) BluePippin size selection considerably increased ONT cDNA read length (top) and optimized recovery of neuronal transcripts, whose length (bottom) exceeds the coverage range of LRS experiments without size selection (gray). (C) Nucleotide composition profile of LRS reads at the 3′ end cleavage site for CIA full-length reads, compared with 3′ ends of discarded reads. (D) Distribution as a function of transcript length of novel and previously annotated isoforms in the CIA transcriptome assembly dataset. (E) Venn diagram showing the number of transcript isoforms (and genes) identified in each tissue in the CIA dataset, scaled by the number of isoforms. Data from different embryonic time points were pooled in (E) and (F). (F) CIA annotation tracks of detected *pumilio* mRNA isoforms in each tissue. Isoforms common to multiple tissues are depicted in gray. Boxes and lines represent exons and introns, respectively. Some introns (dashed lines) are not drawn to scale. TSSs and PASs are represented by arrows and gray stripes, respectively, in the gene model. Replicates per tissue: ONT cDNA: heads, n = 6; embryos 14–16 h, n = 3; embryos 18–20 h, n = 3; ovaries n = 3. FLAM-seq and Iso-seq: heads, n = 3; DRS: heads, n = 1, embryos 14–16 h, n = 3; ovaries, n = 3. See also Figures S1 and S2 and Tables S1–S3.

To examine regulatory links between transcription initiation, exon usage, and APA in *Drosophila*, we first developed a comprehensive LRS isoform annotation approach (Figure 1A). In order to span the maximum range of the coding transcriptome, we used adult brains—the animal tissue with the greatest mRNA isoform diversity and where mRNAs reach their most extreme lengths^35^^,^^36^— as well as embryos at different developmental stages (14–16 and 18–20 h after egg laying [AEL]), and adult ovaries (Table S1). Critically, we size-selected mRNAs (enriching for transcripts >3 kb) using Sage Science BluePippin. We performed Oxford Nanopore Technologies (ONT) cDNA sequencing as well as Pacific Biosciences (PacBio) Iso-seq.^25^ Both LRS approaches use reverse transcription on polyadenylated RNAs and PCR amplification followed by sequencing through a nanopore (ONT cDNA) or single-molecule real-time (SMRT) technology (Iso-seq).

**Figure S1:**
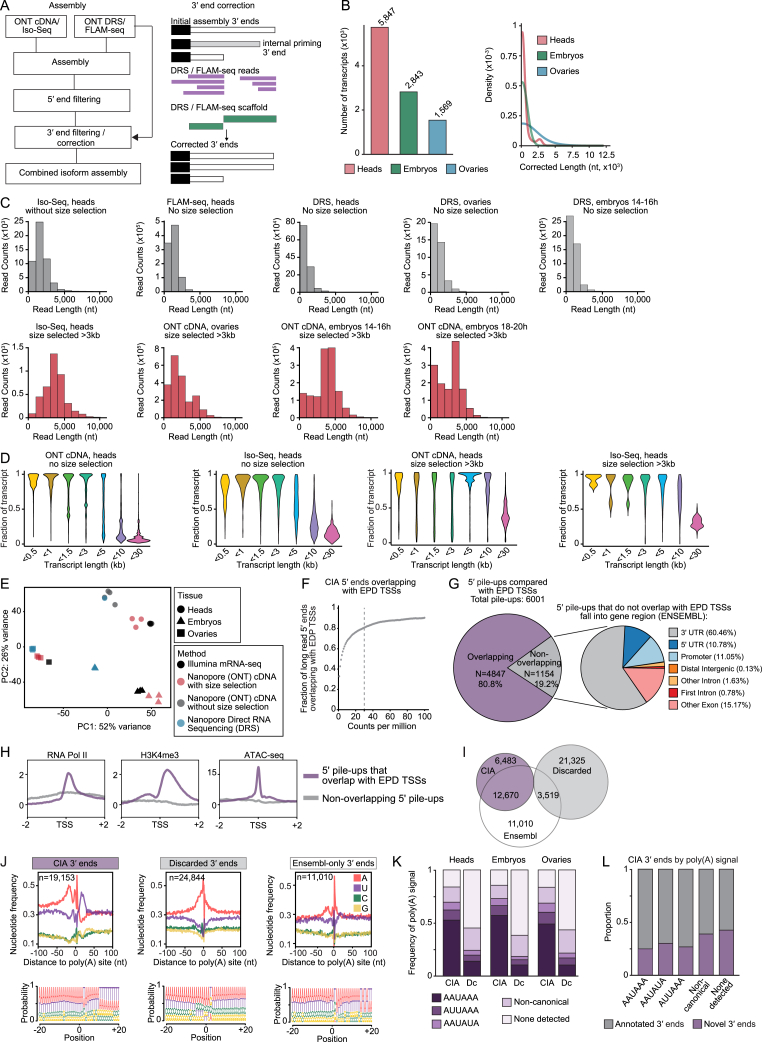
An accurate, comprehensive, full-length *Drosophila* transcriptome, related to Figure 1 (A) Combined isoform assembly (CIA) workflow, and schematic of 3′ end correction and filtering. ONT DRS (in heads: ONT DRS and FLAM-seq) data were used to build a database of confident 3′ ends. The CIA assembly was performed with ONT cDNA and ONT DRS (in heads: ONT cDNA, Iso-seq, ONT DRS, and FLAM-seq) data using FLAIR^40^ and the Eukaryotic Promoter Database (EPD-new).^39^ Note that due to the low number of Iso-seq reads compared with ONT cDNA reads, Iso-seq reads contribute to CIA to a much lower extent. Since this assembly contains 3′ end artifacts, we filtered out any transcripts with 3′ ends not represented in the DRS/FLAM 3′ end database. Assembled transcript models were corrected with DRS/FLAM 3′ ends. (B) Number of corrected transcripts per tissue (left) and average length of correction (right). Data from the two embryo datasets (14–16 h AEL and 18–20 h AEL) were pooled. (C) Read lengths in each tissue with each LRS method. BluePippin size selection (red graphs, below) considerably increased full-length transcript coverage. (D) Full-length transcript coverage per read for nanopore cDNA and PacBio Iso-seq in heads, before (left two graphs) and after (right two graphs) size selection. For each read, the fraction of the target transcript covered is shown; reads were grouped by the length of the target transcript. (E) Principal-component analysis plot of gene expression across the samples (3 biological replicates each) generated using nanopore cDNA with and without size selection, nanopore direct RNA sequencing (DRS), and Illumina short-read mRNA-seq from three tissues. Data from the two embryo datasets (14–16 h AEL and 18–20 h AEL) were pooled. Note that LRS methods without size selection (ONT cDNA and DRS, blue and gray) cluster further from mRNA-seq expression estimates (black) than ONT cDNA with size selection (red). (F) Cumulative plot representing the fraction of long-read 5′ ends that overlap with a TSS described in the Eukaryotic Promoter Database^39^ in a window of 50 nt, as a function of long-read 5′ end counts per million. A 5′ pile-up was defined as a cluster of >30 counts per million per window (dashed line). (G) Pie chart representing the number and proportion of 5′ pile-ups that overlap (purple) with a TSS described in the Eukaryotic Promoter Database.^39^ Non-overlapping pile-ups (gray in the left pie chart) were assessed for the gene region of occurrence (right) as annotated in ENSEMBL. (H) Cumulative enrichment plots of RNA Pol II ChIP-seq signal, H3K4me3 ChIP signal, and ATAC-seq signal detected at 5′ pile-ups (±2 kb) that overlapped (purple) or not (gray) with TSSs annotated in the Eukaryotic Promoter Database. ChIP-seq and ATAC-seq data from *Drosophila* heads are from modENCODE.^61^^,^^49^ (I) Venn diagram describing the overlap of mRNA 3′ ends of LRS reads after filtering (CIA) with filtered-out 3′ ends (discarded) and Ensembl-annotated mRNA 3′ ends. 3′ ends detected by FLAM-seq or DRS represent CIA 3′ends (purple); not-detected 3′ ends (gray) were discarded. (J) Nucleotide composition profiles (spanning 200 nt, top) and sequence logos (spanning 40 nt, bottom) of LRS reads at the cleavage site for each denoted category of 3′ ends from our processing pipeline. Noisy, A-rich distributions are indicative of internal priming. The left and middle panel nucleotide distribution profiles are also shown in Figure 1 and reproduced here for side-by-side comparison with the Ensembl-only category. (K) In each tissue, proportion of 3′ ends at which the indicated poly(A) signals were detected for each category (CIA or discarded [Dc]). Data from the two embryo datasets (14–16 h AEL and 18–20 h AEL) were pooled. (L) In CIA transcripts, proportion of 3′ ends carrying a novel (purple) or a previously annotated (gray) 3′ end. CIA transcripts were categorized by poly(A) signal. Replicates per tissue: ONT cDNA: heads, n = 6; embryos 14–16 h, n = 3; embryos 18–20 h, n = 3; ovaries n = 3. FLAM-seq and Iso-seq: heads, n = 3; DRS: heads, n = 1, embryos 14–16 h, n = 3; ovaries, n = 3. Illumina TrueSeq mRNA-seq: each tissue, n = 2.

Internal priming and RT template switching cause misidentification of 3′ ends in most short-read and LRS approaches.^21^ To avoid these artifacts, we applied ONT direct RNA sequencing (DRS)^37^ and full-length poly(A) and mRNA sequencing (FLAM-seq),^38^ two independent LRS methods that detect the very end of poly(A) tails, and we defined the RNA cleavage site with nucleotide resolution. For a high-precision, high-coverage annotation of *Drosophila* TSSs, we used the Eukaryotic Promoter Database (EPD), a library of RNA polymerase II (RNA Pol II) promoters for which the TSSs were determined experimentally, usually by cap analysis of gene Expression (CAGE) or global run-on (GRO-cap).^39^ We found it crucial to only consider high-quality reads that span entire mRNA isoforms, from 5′ end to 3′ end. We assembled reads from each of the sequencing methods individually using full-length alternative isoform analysis of RNA (FLAIR).^40^ Each assembly was refined to retain only transcripts with a TSS represented in the EPD, and whose 3′ end fell within a FLAM-seq or DRS cluster (Figures 1A, S1A, and S1B), thereby filtering out close to two thirds of all putative transcripts (Tables S1–S3). The remaining transcripts were assembled into a combined isoform assembly (CIA). We detected transcripts with mean read lengths over 4 kb and obtained high full-length coverage of long and ultra-long transcripts typical of the nervous system (Figures 1B, S1C, and S1D). Gene expression estimates from CIA transcripts were highly consistent with those assessed by short-read mRNA-seq in each tissue. In contrast, gene expression estimates assessed from nanopore sequencing on non-size-selected transcripts or DRS displayed substantial deviations from the gold standard method (Figure S1E), showing that size selection, rather than biasing toward longer transcripts, allowed for a better representation of tissue transcriptomes.

To assess the quality of full-length reads, we analyzed CIA 5′ ends and 3′ ends. 5′ end pile-ups of ONT cDNA reads coincided with TSSs annotated in the EPD in 80% of cases; non-overlapping pile-ups fell within distal gene regions, usually 3′ UTRs, and lacked distinctive TSS features such as RNA Pol II ChIP-seq and ATAC-seq peaks (Figures S1F–S1H), indicating high accuracy of *Drosophila* 5′ end annotation in the EPD. CIA 3′ ends harbor the characteristic, defined nucleotide composition^41^ at the cleavage site, whereas filtered-out 3′ ends display noisy A-rich distributions reminiscent of sites of internal priming (Figure 1C). Strikingly, 3′ ends unique to the Ensembl reference globally displayed a noisy nucleotide distribution, indicating that many reference 3′ ends are mis-annotated (Figures S1I–S1L). We conclude that our stringent DRS- and FLAM-seq-guided filtering effectively identified false 3′ ends. Thus, we generated a *Drosophila* mRNA isoform atlas, with 59,970 high-confidence, full-length transcripts. This CIA atlas that represents differential expression and poly(A) tail length of each mRNA isoform in heads, ovaries, and embryos can be accessed at https://hilgerslab.shinyapps.io/ciaTranscriptome.

**Figure S2:**
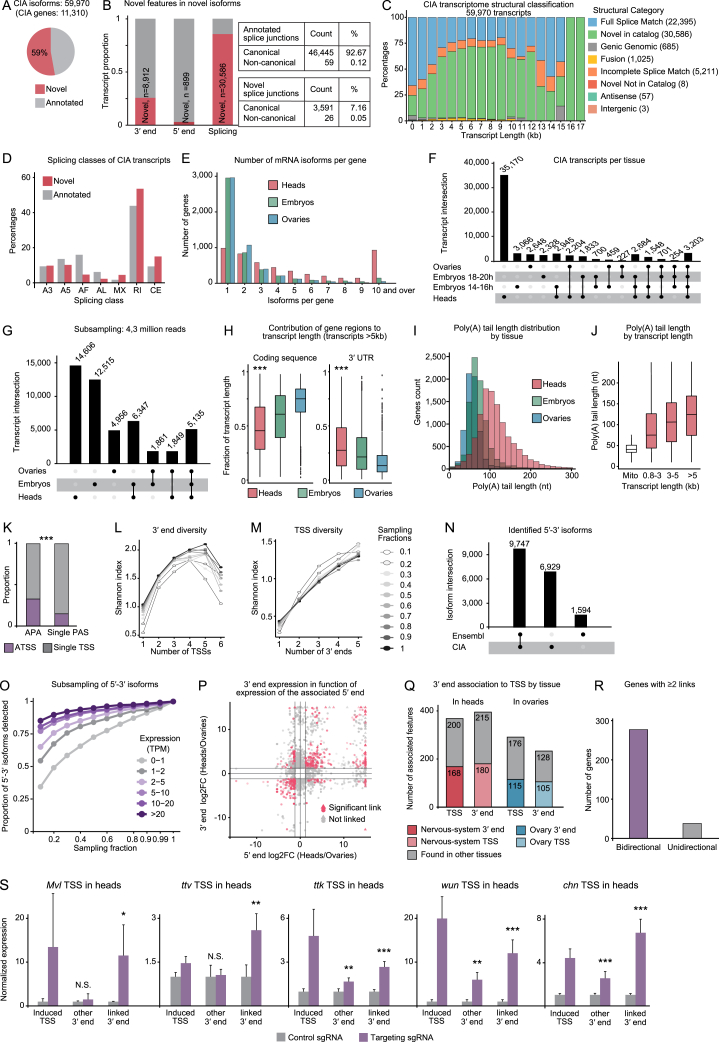
Transcription start sites drive tissue-specific 3′ end expression, related to Figures 1 and 2 (A) Number and proportion of novel (red) and previously annotated (gray) isoforms in the full CIA transcriptome assembly dataset across tissues. (B) Proportion and number of newly identified features (red, novel) that contribute to newly identified isoforms. Most previously unannotated isoforms arise from the differential use of known (annotated) splice junctions with the canonical splice signal and from alternative 3′ end site usage (from 6,483 novel 3′ ends). (C) Proportion of structural and quality annotation of novel transcript isoforms (SQANTI) categories found in the CIA transcriptome assembly as a function of transcript length. The number of transcripts in each category is indicated in parentheses. Transcript lengths were binned by kb. (D) Type and percentage of splicing events found in CIA isoforms. Newly identified isoforms (red) showed no splice class bias, compared with previously annotated isoforms. A3, alternative 3′ splice site; A5, alternative 5′ splice site; AF, alternative first exon; AL, alternative last exon; MX, mutually exclusive exon; RI, retained intron; CE, cassette exon. (E) Number of mRNA isoforms found expressed per gene, in each tissue. (F and G) Overlap of transcript expression across tissues considering all reads from all LRS approaches (F), or sub-sampling 4.3 million ONT cDNA reads (G). Isoforms from 14- to 16-h and 18- to 20-h AEL embryos were pooled in (G). 4.3 million reads were used for subsampling because it represents the smallest read number obtained for an individual tissue (ovaries). Isoforms were considered distinct if they differed by more than 10 nt at exon boundaries, 50 nt at the 5′ end, or 150 nt at the 3′ end. (H) Contribution of coding sequence and 3′ UTR length to transcript length for long (>5 kb) transcripts across tissues. ^∗∗∗^p < 2.2e−16 (ANOVA). Data from the two embryo datasets (14–16 h AEL and 18–20 h AEL) were pooled. (I) Distribution of genes by mean poly(A) tail length for each tissue. Data from the two embryo datasets (14–16 h AEL and 18–22 h AEL) were pooled. (J) Increase in poly(A) tail length as a function of transcript length. Poly(A) tails of mitochondrial mRNAs were included for comparison. (K) Proportion of genes that undergo ATSS in each PAS category. ^∗∗∗^p < 0.001 (two-tailed Fisher’s exact test). (L and M) 3′ end diversity as a function of the number of TSSs per gene (L) and TSS diversity as a function of the number of 3′ ends per gene (M). The Shannon index is a measure of diversity that considers the relative abundance of different species (individual 5′-3′ isoforms) in a population (the sum of all 5′-3′ isoforms). To account for possible coverage biases, the analysis of the whole dataset (black line) was also performed in randomly sampled fractions of the pooled nanopore cDNA data (in grayscale). (N) 5′-3′ isoforms across the Ensembl and CIA reference transcriptomes. 5′-3′ isoforms were considered distinct if they differed by more than 50 nt at the 5′ end or 150 nt at the 3′ end. Comparison after gene expression filtering (>2 transcripts per million [TPM]). (O) Saturation analysis of 5′-3′ isoforms of ATSS-APA genes in the tissue pool, grouped by their expression in transcripts per million (TPM). Reads were randomly sampled in the indicated fractions and the assembly pipeline including 3′ end correction was performed in each fraction. (P) Differential expression of 3′ ends in heads compared with ovaries, plotted as a function of the differential expression of the 5′ end associated with each 3′ end. Red represents 5′-3′ isoforms with a significant 5′-3′ link, i.e., a significant expression change for both the 3′ end and its associated 5′ end (|(log_2_FC)| > 0.5 and adj. p value < 0.05, Wald test, 3 replicates per tissue). (Q) For all tissue-specific TSSs, or 3′ ends, number of associated 3′ ends or TSSs, respectively, that are also tissue-specific. (R) Number of genes with two or more links in which the expression bias is opposite between the two tissues (bidirectional). Significant links were determined as: |log_2_FC (5′-3′ isoform expression)| > 1.5 and adj. p value < 0.01 (Wald test, 3 replicates per tissue). (S) RT-qPCR quantification of the indicated transcript regions in flies in which dCas9-VPR was recruited to nervous-system TSSs for activation (purple). In control flies, dCas9-VPR was co-expressed with a non-targeting sgRNA (gray). Shown are five further genes for which TSS activation was successful in heads (in addition to *Fatp1* shown in Figure 2): *Malvolio* (*Mvl*), *tout-velu* (*ttv*), *tramtrack* (*ttk*), *wunen* (*wun*), and *charlatan* (*chn*). RNA levels were normalized to *RpL32* mRNA, and levels in control flies were set to the value 1. Error bars represent mean ± SD of four biological replicates (five heads per replicate) for each genotype. ^∗^p < 0.05, ^∗∗^p < 0.01, ^∗∗∗^p < 0.001 (one-tailed Student’s t test).

We identified over 30,000 previously undescribed mRNA isoforms. Novel splice variants harbored canonical splicing signals and therefore likely arose from new combinations of known splice sites. In contrast, nearly 9,000 isoforms were characterized by unannotated 3′ end sites (Figures S2A–S2E). Strikingly, isoform novelty drastically increased with transcript length, especially in heads and embryos, two tissues that contain neurons (Figures 1D and S2C), confirming the improved detection of long isoforms of neuronal mRNAs. CIA mRNA isoforms originate from 11,310 genes, 5,020 of which were found to be expressed in all three analyzed tissues. Interestingly, over 80% of these genes are expressed as at least one identical isoform in all three tissues; although most genes expressed in heads were also expressed in other tissues, most CIA isoforms (35,170 out of 59,970) were found exclusively in head samples (Figures 1E, 1F, and S2F). We sequenced neural tissues much more deeply than ovaries and embryos (Table S1), which contributed to, but did not solely account for, the disproportionate representation of brain isoforms (Figure S2G). Our data are consistent with the neural-specific splicing pattern complexity described by modENCODE^35^ and further illuminate the astonishing isoform diversity of the nervous system.

We next investigated ultra-long mRNAs (>5 kb) of the nervous system more closely. Compared with ovaries and embryos, 3′ UTRs disproportionately contribute to transcript length in head tissue (Figure S2H), consistent with the nervous-system-specific 3′ UTR lengthening seen in multiple animal models.^42^^,^^43^^,^^44^^,^^45^ Moreover, nervous system transcripts display surprisingly long poly(A) tails, with their size increasing with transcript length (Figures S2I and S2J). This trend in flies has also been described in human cells and *C. elegans*,^38^ and it suggests a conserved coupling between distal PAS selection and tail length, possibly reflecting the result of distinct turnover kinetics and a potential role for long poly(A) tails in the protection of ultra-long transcripts.

### Coupling between transcript 5′ ends and 3′ ends

**Figure 2:**
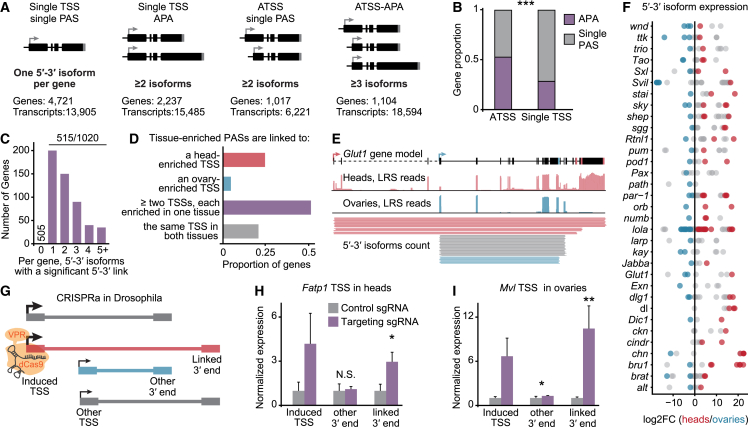
Transcription start sites drive tissue-specific 3′ end expression (A) Gene categorization according to TSSs and PASs detected in CIA full-length isoforms. 5′-3′ isoforms were considered distinct if they differed by more than 50 nt at the 5′ end or 150 nt at the 3′ end. The use of several TSSs (arrows) and PASs (stripes) characterizes ATSS and APA genes, respectively. (B) Proportion of genes that undergo APA in each TSS category. ^∗∗∗^p < 0.001 (two-tailed Fisher’s exact test). (C) Number of 5′-3′ isoforms that show a significant difference in expression between heads and ovaries per gene, for all 1,020 ATSS-APA genes expressed in both tissues. Significant links were determined as: |log_2_FC (5′-3′ isoform expression)| > 1.5 and adj. p value < 0.01 (Wald test, 3 replicates per tissue). (D) Proportion of genes in which 3′ ends that are enriched in one tissue over the other are associated with a TSS enriched in the same tissue (|log_2_FC| > 0.5 and adj. p value < 0.05, Wald test, 3 replicates per tissue). Purple indicates that enrichment occurs in both tissues (bidirectional) for at least two PASs. (E) *Glut1* genomic alignment coverage tracks for long reads from heads and ovaries, and depiction of full-length reads representing distinct 5′-3′ isoforms. Read counts per isoform are shown to scale, but each line represents multiple reads. Significant 5′-3′ links are colored in red (heads) and blue (ovaries). Isoforms represented in gray are found in both tissues. Some introns (dashed lines) are not drawn to scale. (F) Differential isoform expression in *Drosophila* heads compared with ovaries for a panel of genes that display bidirectional 5′-3′ isoform regulation. Isoforms with a significant 5′-3′ link are colored blue (ovary link) and red (head link). (G) CRISPRa in fly tissues. Each fly expresses sgRNAs complementary to a tissue-enriched TSS. Association of the sgRNA with a co-expressed dCas9 protein, fused with the transcriptional activator VPR, induces gene activation at the target TSS (red). 5′-3′ isoforms are represented as boxes joined by a straight line; significant links are colored. (H and I) RT-qPCR quantification of the indicated transcript regions in flies in which dCas9-VPR was recruited to nervous-system TSSs for activation (purple). In control flies, dCas9-VPR was co-expressed with a non-targeting sgRNA (gray). TSS activation of two representative genes, *Fatty acid transport protein 1* (*Fatp1*) and *Malvolio* (*Mvl*) is shown in heads (H) and ovaries (I). RNA levels were normalized to *RpL32* mRNA, and levels in control flies were set to the value 1. Error bars represent mean ± SD of four biological replicates for each genotype and tissue. ^∗^p < 0.05 and ^∗∗^p < 0.01 (one-tailed Student’s t test). See also Figure S2 and Tables S4 and S5.

The CIA transcriptome now allows us to quantify the co-occurrence of distinct co-transcriptional events in full-length mRNA isoforms. We focused on the analysis of regulatory relationships between transcription initiation and transcription termination. First, we categorized genes based on the number of identified TSSs and PASs in the CIA dataset (Figure 2A). We found that genes with alternative TSS usage (ATSS) undergo APA disproportionately often, and vice versa (Figures 2B and S2K); moreover, 3′ end diversity increases as a function of TSS number, and vice versa (Figures S2L and S2M). This could suggest that ATSSs have evolved to drive the production of distinct 3′ ends. To study couplings between TSSs and PASs, we quantified the differential use of 3′ ends as a function of the 5′ end with which they are associated. We term a “5′-3′ isoform,” a combination of 5′ end and 3′ end, i.e., a co-occurrence of any 5′ end and 3′ end in the same full-length CIA transcript. Importantly, many of the 5′-3′ isoforms we detected in our sensitive LRS approach may have resulted from unproductive transcription and represent “noise” rather than biologically relevant isoforms. To eliminate these isoforms, we used an expression cutoff of >2 transcripts per million (TPM). We found over 16,000 5′-3′ isoforms, almost 7,000 of which were novel (Figure S2N). We subsampled ONT cDNA reads and assessed the number of identified 5′-3′ isoforms for each fraction and for different expression categories. Above cutoff, we reached near-saturation of 5′-3′ isoform detection, even for genes with multiple TSSs and multiple PASs (ATSS-APA genes) (Figure S2O), strongly suggesting that our analysis faithfully represents the 5′-3′ isoform landscape in *Drosophila* tissues.

### TSSs drive the selection of tissue-enriched 3′ end sites

To assess whether APA is driven by the use of distinct TSSs, we first asked whether tissue-specific 3′ end expression is associated with tissue-specific 5′ ends. Ovaries and heads constitute the two tissues at the extremes of the APA spectrum, with shifts toward proximal and distal PAS selection, respectively.^43^ We calculated differential 3′ end and 5′ end expression between the two tissues to identify “nervous-system 3′ ends” and “ovary 3′ ends,” and we then assessed differential 5′-3′ isoform expression in genes expressed in both tissues (Figure S2P). We discovered that for over half of all ATSS-APA genes, at least one 5′-3′ isoform is enriched in one tissue compared with the other, representing a significant 5′-3′ link (Figure 2C; Table S4), and distinct TSSs are specifically associated with 3′ ends with differential expression between the two tissues (Figure 2D). Moreover, almost half of all nervous-system 3′ ends were specifically expressed from a nervous-system TSS, and vice versa (Figure S2Q). In genes with several significant 5′-3′ links, we observe, almost always, a pattern of bidirectionality in which one 5′-3′ isoform is enriched in heads while the other is enriched in ovaries (Figures 2E, 2F, and S2R). Our results show that ovary- and head-specific PAS usage is linked to the alternative use of TSSs and suggest that TSSs influence PAS selection.

To functionally test this hypothesis *in vivo*, we used the CRISPR transcriptional activator (CRISPRa) system, in which a catalytically dead Cas9 (dCas9) fused to the VPR activator domain can be recruited to the upstream TSSs of individual genes by single-guide RNAs (sgRNAs).^46^ We tested all “bidirectional” genes for which a sgRNA strain was available (53 genes) and in which the upstream TSS was head- or ovary-enriched (23 TSSs, Figure 2G; Table S5). TSS activation failed in ovaries for all tested sgRNAs except one; in heads, we obtained significant gene activation for six nervous-system TSSs. In all cases, activation of the nervous-system TSS caused a specific increase in the expression of the linked, nervous-system 3′ end (Figures 2H and S2S). Notably, induction of the *Malvolio* (*Mvl*) nervous-system TSS in ovaries caused the ectopic expression of the linked, nervous-system 3′ end, demonstrating that specific TSS activation is sufficient to drive atypical 5′-3′ isoform expression (Figure 2I). Our data thus show that the site of transcription initiation drives head-specific 3′ end site usage, thereby crucially contributing to the establishment of the distinct 3′ UTR landscape of the nervous system.

### Co-expression of multiple 5′-3′ isoforms in neuronal cell types

**Figure S3:**
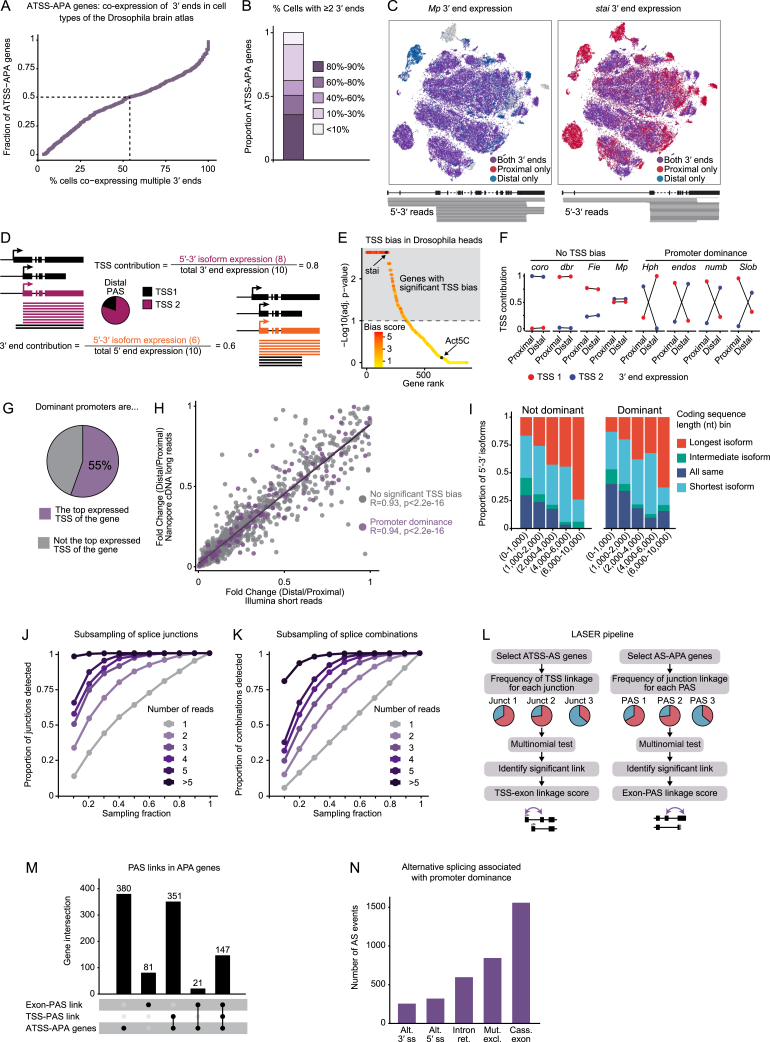
Dominant promoters drive PAS choices, related to Figure 3 (A) Cumulative plot representing the fraction of ATSS-APA genes as a function of the fraction of cells co-expressing more than one 3′ end across cell types with an expression of more than 0.1 normalized counts. (B) Proportion of ATSS-APA genes in which two or more isoforms were found to be expressed in the indicated percentage of cells in single-cell RNA-seq data from the Drosophila Brain Atlas.^49^ For most genes (above the 0.5 proportion), most (over 50%) cells express two or more 3′ ends. (C) t-distributed stochastic neighbor embedding (t-SNE) maps representing 3′ end expression in *Drosophila* brain cell types for the two representative genes Multiplexin (Mp) and *stathmin* (*stai*). Cells are colored according to their expression of either only the proximal (red), only the distal (blue), or both 3′ end isoforms (purple). Shown below are the gene model and 5′-3′ reads representing the expression of the detected 5′-3′ isoforms. Some introns (dashed lines in the gene model) are not drawn to scale. (D) Schematic representation of how TSS and 3′ end contributions to 5′-3′ isoform expression were calculated. Full-length 5′-3′ reads were quantified and assigned to 5′-3′ isoforms. For a given 3′ end, the contribution of each 5′-3′ isoform to the expression of the 3′ end was calculated (pink), as well as for a given TSS, the contribution of each 5′-3′ isoform to the expression of the 5′ end (orange). A TSS is termed a dominant promoter for a 3′ end if the respective 5′-3′ isoform expression has a contribution to 3′ end expression significantly higher (p < 0.1, chi-squared test with Monte Carlo simulation and Benjamini-Hochberg correction, also see E) than that of all other 5′-3′ isoforms for the same 3′ end. (E) TSS bias in ATSS-APA genes assessed using multinomial testing in *Drosophila* heads. The observed vs. expected counts of 5′-3′ isoforms were used for multinomial testing (chi-squared test with Monte Carlo simulation and Benjamini-Hochberg correction, n = 3). Genes are represented as dots, ranked by p value and color-coded according to bias score (promoter dominance score: absolute value of residuals). Highest-ranked genes (220 genes in the brain) represent near-exclusive 5′-3′ combinations, as exemplified by *stai*. (F) Promoter dominance and absence thereof (no TSS bias) shown on representative ATSS-APA genes with two TSSs and two PASs. The proportional contribution of the first TSS (red) and the second TSS (blue) to the expression of the proximal and the distal 3′ end of the same gene are indicated. Lines crossing signify TSS contributions that differ significantly between the PASs. (G) Pie chart representing the percentage of dominant promoters that constitute the top expressed TSS of the gene in heads. (H) Scatterplot showing the expression ratio between isoforms expressing the distal and proximal PAS, respectively, measured by long-read sequencing (ONT cDNA), in function of ratios measured by mRNA-seq (Illumina short reads). The ratios were calculated by estimating the ratio of normalized TPM (transcripts per million) assigned to proximal and distal 3′ ends in APA genes. Each dot represents a gene. The correlation coefficient (two-tailed Pearson correlation) is indicated for genes with a dominant promoter (promoter dominance) and TSS-unbiased genes (no significant TSS bias). (I) Proportion of 5′-3′ isoforms by category, expressing the indicated types of coding sequence, as a function of coding sequence length. Coding sequences are categorized by length within the gene context and represent either the longest, shortest, or an intermediate CDS isoform. Coding sequences of a gene were considered of identical length (all same) if none differed by more than 200 nt. 5′-3′ isoforms are grouped into 5′-3 isoforms with a dominant promoter (dominant) and 5′-3′ isoforms with no dominant promoter (not dominant). (J and K) Saturation analysis of splice junctions (J) and splice combinations (K) in CIA transcripts, grouped by their expression in number of reads. Reads were randomly sampled in the indicated fractions and a junctions (J) or combinations (K) database was built for each fraction. Splice junctions are exon-exon junctions. Splice combinations are unique assemblies of consecutive exons for each gene. Exons containing, or upstream of, a TSS (first exon), or containing or downstream of a PAS (last exon), were excluded from the analysis. (L) Long-reads-based alternative splicing estimation and recognition (LASER) framework to identify TSS biases in alternatively spliced (AS) genes (left), and splicing biases in alternatively polyadenylated (APA) genes (right). TSS-exon bias: for each splice junction of each ATSS-AS gene, the observed vs. expected frequencies of TSS-junction combinations were calculated to identify TSSs disproportionately associated with the junction (TSS-exon links). Exon-PAS bias: for each PAS of each AS-APA gene, the observed vs. expected frequencies of splice junction-PAS combination were calculated to identify splice junctions disproportionately associated with the PAS (exon-PAS links). Significant TSS-exon and exon-PAS links were identified by multinomial testing (p < 0.1, chi-squared test with Monte Carlo simulation and Benjamini-Hochberg correction) and assigned a linkage score (sum of squares of residuals). Splice junctions are exon-exon junctions. (M) Genes in which alternative polyadenylation is linked to alternative splicing (exon-PAS links) or transcription start sites (TSS-PAS link: promoter dominance), or both. Intersections between the gene sets are depicted as connecting lines. The number of genes in each exclusive group is indicated. Only 81 genes with an exon-PAS link were identified outside of the ATSS-APA gene group, and only 21 within the ATSS-APA gene group that were not associated with a dominant promoter (TSS-PAS link). (N) Type and number of alternative splicing events found in mRNA isoforms transcribed from dominant promoters: alternative 3′ splice site; alternative 5′ splice site; intron retention; mutually exclusive exon; cassette exon.

The coordination between tissue-specific TSSs and APA could be mediated by tissue-specific *trans*-factors; for example, the pan-neuronal RBP ELAV promotes APA of individual genes in a TSS-dependent manner.^11^ To explore the regulation of co-transcriptional processing independently of the cellular environment, we investigated 5′-3′ links at the gene level in a single tissue—the brain—in which ATSS and APA are particularly abundant. Since APA isoform usage displays cell-to-cell heterogeneity,^47^ and some 3′ ends can be specific to certain cell populations,^48^ we assessed whether the 5′-3′ links that we identified in *Drosophila* heads tend to be expressed in the same cell, or whether on the contrary, distinct isoforms are exclusive to different cell types. Using the *Drosophila* brain atlas,^49^ we evaluated every CIA 3′ end at the single-cell level and quantified the co-occurrence of different 3′ ends of the same gene in each of the 177 cell types described in the dataset. We found that the majority of ATSS-APA genes are co-expressed as several APA isoforms in most cell types, and we did not detect a general trend of mutually exclusive 3′ end isoform expression within the brain (Figures S3A–S3C). We conclude that differential usage of TSSs and PASs can occur within the same cell type, independently of tissue-specific or cell-type-specific factors. Hence, we can use the nervous system 5′-3′ isoform dataset to probe PAS preference within the same cell populations.

### Global bias of 3′ end site selection depending on the TSS

**Figure 3:**
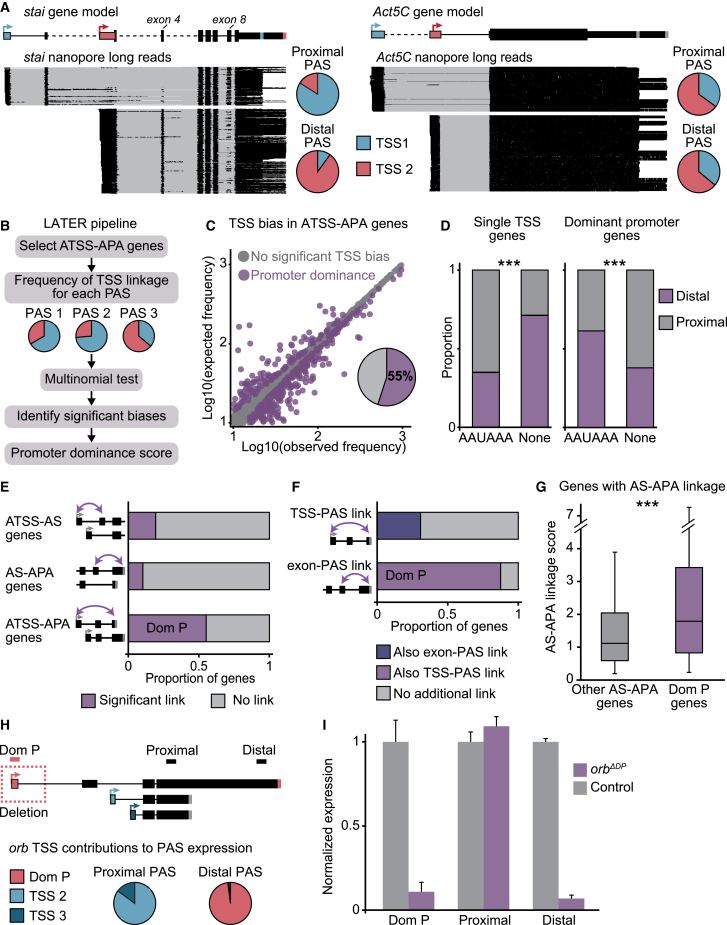
Dominant promoters drive PAS choices (A) Representative examples of ATSS genes with promoter dominance (*stai*, left) and no TSS bias (*Act5C*, right). Nanopore full-length reads (black) are shown below the gene models. Pie charts represent the contributions of each TSS to the expression of each 3′ end. PASs subjected to promoter dominance are represented as stripes in the color of their respective linked TSS. Some introns (dashed lines) are not drawn to scale. (B) LATER framework. For each PAS of each ATSS-APA gene, the observed vs. expected frequencies of 5′-3′ isoforms were calculated to identify TSSs that disproportionately contribute to PAS expression (promoter dominance). (C) Expected frequencies of 5′-3′ isoforms shown as a function of the frequencies measured for each PAS in heads. Significant 5′-3′ isoforms by multinomial testing (p < 0.1, chi-squared test with Monte Carlo simulation and Benjamini-Hochberg correction, 3 replicates, pooled) are represented as purple dots (promoter dominance); isoforms with no significant TSS bias are in gray. (D) PAS usage when either the canonical (AAUAAA) or no detected (none) poly(A) signal is found within a 50-nt window of the most proximal PAS of the gene, in APA genes with a single promoter (left) and in APA-ATSS genes with a dominant promoter (right). Proximal and distal denote PASs located in the proximal 20% or distal 80% of the 3′ UTR, respectively. ^∗∗∗^p < 0.001 (two-tailed Fisher’s exact test). (E) Proportion of genes in each category displaying a significant TSS-exon link (top, assessed by LASER), exon-PAS link (middle, LASER), or TSS-PAS link (promoter dominance, bottom, LATER). (F) Proportion of ATSS-APA genes with TSS-PAS links that also exhibit at least one exon-PAS link, and vice versa. (G) Strength of AS-APA links in presence (Dom P) and absence (other) of a dominant promoter. The linkage score corresponds to the sum of squares of residuals (×10^2^) from the LASER analysis. ^∗∗∗^p = 6.3e−11 (two-tailed Student’s t test). (H) Schematic of *orb* mRNA isoforms. In *orb*^*ΔDP*^ flies, the region surrounding the dominant promoter was deleted (dashed box). Pie charts show the contribution of each TSS to the expression of each 3′ end in wild-type flies. (I) RT-qPCR quantification of the indicated transcript regions in *orb*^*ΔDP*^ and control embryos (18–20 h AEL). RNA levels were normalized to *orb* coding sequence (CDS), and levels in control flies were set to the value 1. Error bars represent mean ± SD of three biological replicates for each genotype. Control flies are progeny of a non-mutated sibling of the parental *orb*^*ΔDP*^ fly. See also Figure S3 and Table S4.

The identification of full-length gene isoforms of ATSS-APA genes in heads revealed that in many cases (e.g., *stai*), distinct PASs were preferentially associated with specific TSSs, while for other genes (e.g., *Act5C*) there was no such bias (Figure 3A). We set out to assess whether the competitive use of PASs is regulated at the site of transcription start. To discern regulatory links transcriptome-wide between transcription start and 3′ end formation, we developed the computational framework long-reads-based alternative termination estimation and recognition (LATER) (Figure 3B). For all ATSS-APA genes, for a given PAS, we calculated the frequency of association of each TSS with the expression of the associated 3′ end (Figures 3A, 3B, and S3D). We defined two modes of 3′ end site selection in ATSS-APA genes: “TSS-unbiased,” in which the association frequencies of distinct TSSs with a given 3′ end did not significantly differ; and “promoter dominance,” in which one TSS was disproportionately associated with the expression of a specific 3′ end. Strikingly, deviations from the expected proportions were the rule rather than the exception, with most (55%) ATSS-APA genes displaying promoter dominance in at least one tissue (Figures 3C, S3E, and S3F; Table S4).

Highly expressed genes displayed predominantly short 3′ UTRs, and stronger promoters were found to favor the selection of proximal PASs in reporter assays,^50^ consistent with the idea that high transcriptional activity enhances 3′ end processing on a first-come, first-served basis.^51^ In contrast, a fast RNA Pol II elongation rate correlates with the use of more distal PAS in yeast.^52^ However, we did not observe any significant difference in expression levels of isoforms from our identified dominant promoters (Figure S3G); importantly, full-length 5′-3′ isoform detection and categorization as dominant-promoter-isoform were not biased by read length for transcripts up to 10 kb long (Figures S3H and S3I). Therefore, transcript length or TSS strength cannot explain PAS selection in cases of promoter dominance. With the ability to quantitatively assess individual 5′-3′ isoforms, we demonstrate a global effect of TSS selection on differential 3′ end expression, causally linking transcription initiation to termination.

### Dominant promoters override strong poly(A) signals and constrain AS

We asked whether dominant promoters showed a propensity to override well-defined rules of mRNA processing. For APA genes, differential 3′ end expression is thought to depend on PAS “strength”: unless specifically inhibited in *trans*, PASs containing the hexamer AAUAAA and variants thereof are rarely bypassed to produce a more distal 3′ end.^53^^,^^54^ For APA genes with a single promoter, the presence of the AAUAAA sequence was indeed a predictor of proximal PAS usage in our dataset, and skipping of the proximal PAS usually occurred in the absence of a poly(A) signal. Strikingly, ATSS-APA genes with dominant promoters showed the opposite trend; in fact, proximal PASs containing AAUAAA were preferentially skipped in transcripts arising from a dominant promoter (Figure 3D).

Next, we tested whether splicing plays a role in the observed 5′-3′ couplings, possibly representing the regulatory intermediate between dominant promoters and 3′ end site selection. First, we ensured that splice isoform coverage in long reads was sufficient to assess exon-exon junction choice. Except for isoforms identified with one single read, likely representing very rare or aberrant variants, we reached saturation of splice isoform detection (Figures S3J and S3K). We developed long-reads-based AS estimation and recognition (LASER), based on the same principles as LATER (Figure S3L), to identify disproportionate association frequencies between distinct TSSs and exon-exon junctions—“TSS-exon links”—as well as between exon-exon junctions and PASs—“exon-PAS links.” Compared with TSS-PAS links (promoter dominance), we identified surprisingly little coupling between AS and APA, with significant links in about 10% of AS-APA genes (Figures 3E, S3M, and S3N; Table S4). A significant link between AS and 3′ end site selection was seen in about one-third of genes with a dominant promoter; for example, *stai* exons 4 and 8 are near-mutually exclusively associated with distinct PASs and their respective dominant promoters (Figures 3A and 3F). This enrichment, but lack of systematic association of AS with APA led us to hypothesize that exon-PAS couplings are a consequence, not a causal intermediate, of the influence of dominant promoters on co-transcriptional processing. Indeed, we find that in ATSS-APA genes, exon-PAS links almost always (88%) occur when transcription starts from a dominant promoter. Moreover, exon-PAS links are significantly weaker in the absence of a dominant promoter (Figures 3F and 3G). We conclude that in ATSS-APA genes, AS does not represent a necessary intermediate step for biased 3′ end selection by dominant promoters, although it may influence APA in individual cases. Together, our findings indicate that sites of transcription initiation direct APA independently of poly(A) signal strength and also impose a constraint on other RNA processing events such as splicing.

To functionally validate 5′-3′ links and verify that 3′ end choice is mediated by dominant promoters *in vivo*, we generated the fly mutant *orb*^*ΔDP*^, in which the dominant promoter of the gene *orb* was specifically deleted by CRISPR-Cas9-mediated gene editing. *Orb* possesses two 3′ ends and three TSSs, with the first TSS dominantly associated with the distal-most 3′ end (Figure 3H). In *orb*^*ΔDP*^ embryos, expression of the distal but not the proximal 3′ end was massively depleted (Figure 3I). Our data thus show that dominant promoters influence PAS selection and can mediate skipping of canonical poly(A) signals to favor more distal sites of transcription termination.

### 3′ end site selection through promoter dominance impacts transcriptome identity and gene function

**Figure 4:**
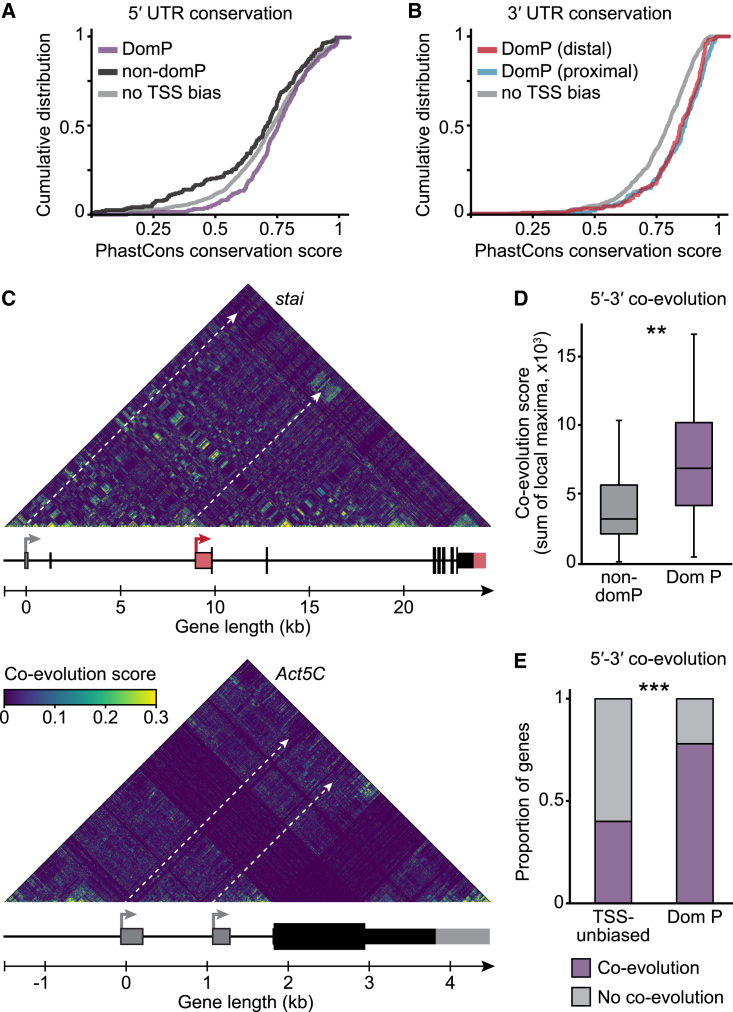
Functional impact of promoter dominance on transcriptome diversity and tissue identity (A) Cumulative distribution of PhastCons conservation scores for 5′ UTRs transcribed from dominant promoters (DomP), other 5′ UTRs of dominant-promoter genes (non-domP), and 5′ UTRs of TSS-unbiased genes. (B) Cumulative distribution of PhastCons scores for 3′ UTR sequences generated through the use of PASs linked to dominant promoters (DomP) and in genes with no TSS bias. 3′ UTR sequences upstream (DomP proximal) and downstream (distal) of the proximal PAS, and the entire 3′ UTR (no TSS bias), were used for the analysis. (C) Maps of co-evolved nucleotides, in all-by-all comparisons, in the genomic regions of *stai* and *Act5C*. In the grid, the normalized mutual information (co-evolution score) is represented in color for each position pair. Dashed arrows indicate regions of comparison between promoter-proximal sequences and distal 3′ UTRs. Dominant promoters and linked 3′ UTR sequences are in red. (D) Co-evolution scored for dominant (DomP) compared with non-dominant (non-domP) promoters. ^∗∗^p = 0.0037 (two-tailed Student’s t test). The co-evolution score for each TSS was calculated as the sum of co-evolution score maxima in a matrix region comparing the regions TSS − 1 kb and 3′ UTR (entire sequence). (E) Proportion of genes with (Dom P) or without (TSS-unbiased) promoter dominance that display co-evolution for at least one TSS. ^∗∗∗^p = 0.0002112 (two-tailed Fisher’s exact test). A TSS was considered to display co-evolution if the TSS’s co-evolution score was in the top 50% quartile of all TSS scores. In (D) and (E), all TSSs of 100 ATSS-APA genes were scored by promoter dominance p value, the top 50 (DomP), and bottom 50 (TSS-unbiased) genes. See also Figure S4.

To assess the functional importance of the reported connection between TSSs and PASs, we first sought to determine if it is evolutionarily conserved. We found that 5′ UTRs transcribed from dominant promoters and 3′ UTR sequences generated via dominant-promoter-associated PASs (“dominant-promoter-3ʹ UTRs”) were more conserved than their non-dominant and unlinked counterparts, respectively (Figures 4A and 4B). Following the notion that functional interactions can be detected through evolutionary couplings,^55^ we performed a mutual information analysis^56^ to test whether 3′ end site regions and their dominant promoters mutate jointly to maintain genetic interactions. We calculated the co-evolution score for each pair of nucleotide positions within the gene *stai*. Strikingly, a cluster of high-scoring nucleotide pairs could be identified between 3′ UTR sequences and regulatory regions upstream of the linked dominant promoter, but not the non-dominant promoter. *Act5C*, a gene with no TSS bias, did not display any 5′-3′ co-evolution clusters (Figure 4C). We performed a more global analysis, selecting 100 ATSS-APA genes (top and bottom 50 by promoter dominance p value), and scored, for each gene, co-evolution clusters in nucleotide pair matrices between 5′ end regions (TSS − 1 kb) and the 3′ end region (3′ UTR). We found that co-evolution scores were significantly higher for dominant promoters, compared with other TSSs; most dominant-promoter genes, but not TSS-unbiased genes, showed strong co-evolution between 5′ end and associated 3′ end sequences (Figures 4D and 4E). Our results show not only that sequences generated directly (5′ UTRs) or indirectly (linked 3′ UTRs) from dominant promoters are conserved but also that evolutionary pressure maintains the link between them.

**Figure S4:**
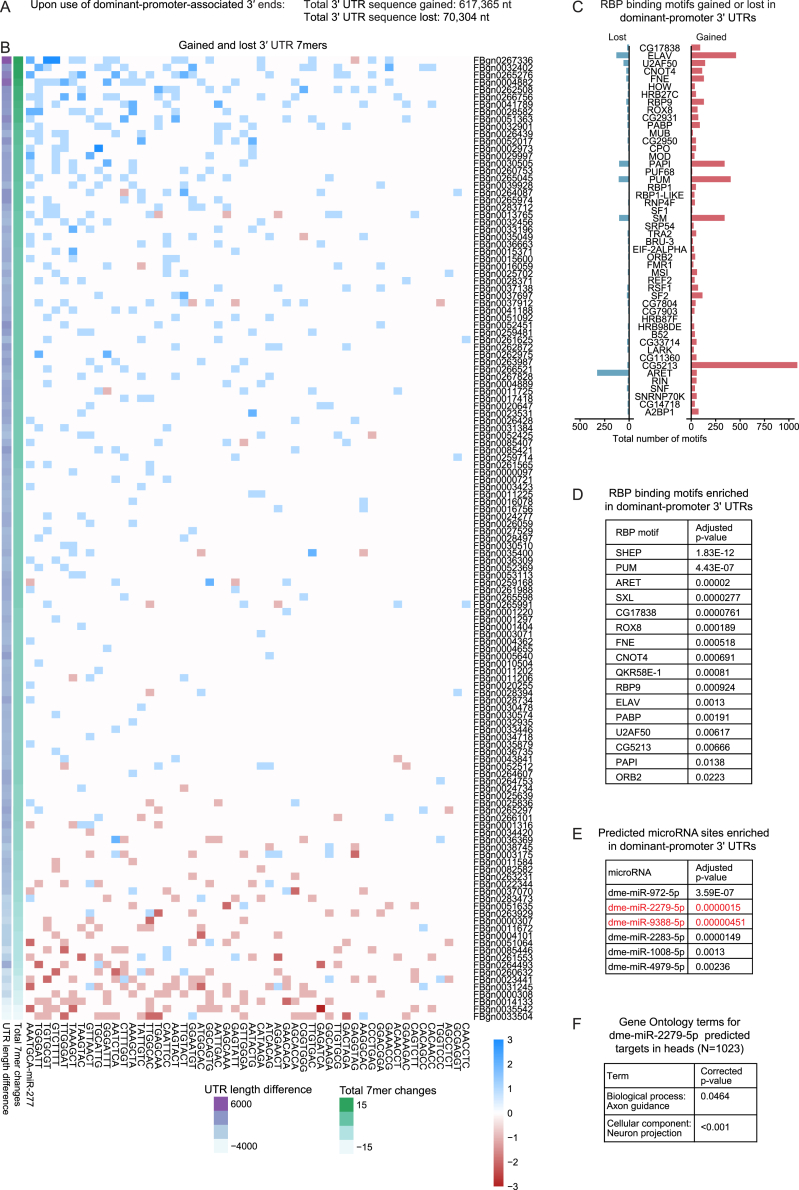
Functional impact of promoter dominance on transcriptome diversity and tissue identity, related to Figure 4 (A) 3′ UTR sequence length gained or lost by the predicted shift in PAS selection as a result of promoter dominance, for 173 dominant-promoter genes, in fly heads. “Gained” and “lost” refers to dominant-promoter-3′ UTRs associated with the distal and proximal PAS, respectively. (B) Number of potential binding sites (7-mers) gained (blue) or lost (red) by the predicted shift in PAS selection as a result of promoter dominance, for a set of 65 highly conserved and highly expressed microRNAs (collapsed into 52 seed sequences), for 173 dominant-promoter genes, in fly heads. The total number of gained and lost sites and the 3′ UTR length difference between proximal and distal isoforms are indicated at the bottom. (C) Number of binding motifs for the indicated RNA-binding proteins (RBPs) gained or lost by the predicted shift in PAS selection as a result of promoter dominance, for 173 dominant-promoter genes, in fly heads. (D) RBP binding motifs enriched in dominant-promoter 3′ UTRs associated with the distal PAS, compared with 3′ UTRs associated with the proximal PAS. (E) Predicted microRNA binding sites enriched in dominant-promoter 3′ UTRs associated with the distal PAS, compared with 3′ UTRs associated with the proximal PAS. MicroRNAs detected in *Drosophila* heads (MirGeneDB v2.1)^57^ are shown in red. (F) Enriched gene ontology terms in 1,023 mRNAs expressed in heads that are predicted targets for dme-miR-2279-5p.

We next computationally predicted the consequence of disrupting TSS-PAS links and the ensuing 3′ end mis-selection. In *Drosophila* heads, differential 3′ end site selection by dominant promoters results in a change in protein-CDS, 3′ UTR lengthening, and 3′ UTR shortening in 40%, 42%, and 18% of cases, respectively. A substantial amount of regulatory 3′ UTR sequence is gained through dominant-promoter-mediated 3′ UTR lengthening (Figure S4A); we sought to quantify the influence of dominant promoters by computing the occurrence, in either 3′ UTR isoform, of potential binding sites for neuronal RBPs and microRNAs highly conserved and enriched in fly heads, since these are more likely to exert a functionally relevant effect on target mRNAs.^57^ Interestingly, binding motifs for miR-277, a microRNA involved in synaptogenesis with a possible role in neurodegeneration,^58^^,^^59^ were the most impacted by dominant-promoter-mediated 3′ UTR lengthening (Figure S4B). In addition, dominant-promoter 3′ UTRs were enriched in putative binding sites for RBPs well known for specialized neuronal roles, such as pumilio (Pum) and alan shepard (Shep), as well as for miR-2279, a poorly expressed and conserved microRNA that is nonetheless predicted to target neural pathways related to axonal projections (Figures S4C–S4F). This indicates that dominant-promoter-associated 3′ UTR sequences function in the regulation of the encoded protein in an isoform-specific manner; our analyses predict that disruption of conserved TSS-PAS links causes a widespread mis-selection of 3′ end sites, resulting in loss of tissue-specific protein isoforms and 3′ UTR-mediated regulation by microRNAs and RBPs, strongly suggesting that regulation through dominant promoters is functionally relevant for animal fitness.

### A combination of epigenetic features defines the chromatin environment of dominant promoters

**Figure S5:**
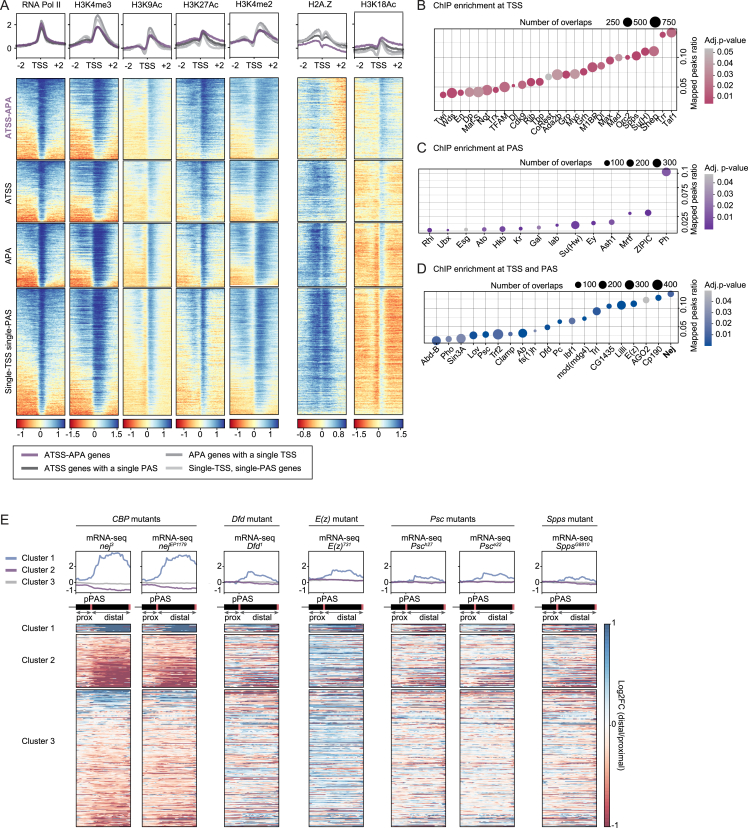
TSSs exert promoter dominance through specific chromatin signatures, related to Figure 5 (A) Heatmaps and cumulative enrichment plots of ChIP-seq signal at TSS ± 2 kb for RNA Pol II, the histone marks H3K4me3, H3K9Ac (typical for active promoters and TSSs of expressed genes), H3K27Ac, and H3K4me2 (active enhancer and TSS marks), the histone variant H2A.Z and the histone mark H3K18Ac genome-wide. Genes are grouped by CIA categories. ChIP-seq data from *Drosophila* heads are from modENCODE.^61^ (B–D) ChIP-seq peak enrichment analysis at the TSS and PAS of dominant promoter isoforms. Represented are factors significantly enriched (adj. p value < 0.05) at either the TSS ± 150 nt (B), the associated PAS ± 150 nt (C), and both (D), ranked by the ratio of total peaks that map to the TSS (B and D) or PAS (C). (E) Enrichment (blue) and depletion (red) of distal 3′ UTR RNA expression in the indicated mutants compared with control embryos. mRNA-seq heatmaps and profile plots display 0.5 kb upstream of the proximal poly(A) site (prox), and the distal 3′ UTR downstream (distal, scaled region). For *CBP* and *Psc*, results from two independent mutant alleles are shown. RNA was obtained from hand-sorted embryos at 16–18 h AEL in three biological replicates for each genotype (except *CBP* mutants: 14–16 h AEL, four replicates). Genes are grouped into three clusters by k-means clustering using both CBP ChIP-seq signal at proximal PASs and mRNA-seq signal in *nej*^3^ and *nej*^*EP1179*^ mutants. Heatmaps for *CBP* mutants, also shown in Figure 5, are reproduced here with a different color scale for side-by-side comparison with the other mutants. CBP, Dfd, E(z), and Psc were found enriched at the TSS of dominant promoters and their associated 3′ end; Spps was found enriched at the TSS of dominant promoters only.

One possible interpretation of the observed 5′-3′ coupling is that dominant promoters possess a characteristic that subjects the nascent transcript to modified rules of co-transcriptional processing. Splicing and 3′ end cleavage have been shown to be influenced by the presence of particular chromatin elements at the sites of transcription initiation and termination, respectively.^60^ We set out to identify whether dominant promoters possess a common regulatory feature that mediates coupling between TSS and PAS. We analyzed ChIP-seq data generated in *Drosophila* heads (modENCODE^61^) to assess the *in vivo* location of over 40 histone marks, histone variants, and transcription factor binding sites. We found that promoter regions of ATSS-APA genes, while not displaying any notable enrichment in RNA Pol II or common repressive or active chromatin marks, were strongly depleted for the histone variant H2A.Z. Conversely, acetylation of histone H3 at lysine 18 (H3K18Ac), a histone mark associated with gene activation and transcriptional priming in developmental transitions,^62^ was specifically enriched around the TSS of ATSS-APA genes (Figure S5A).

**Figure 5:**
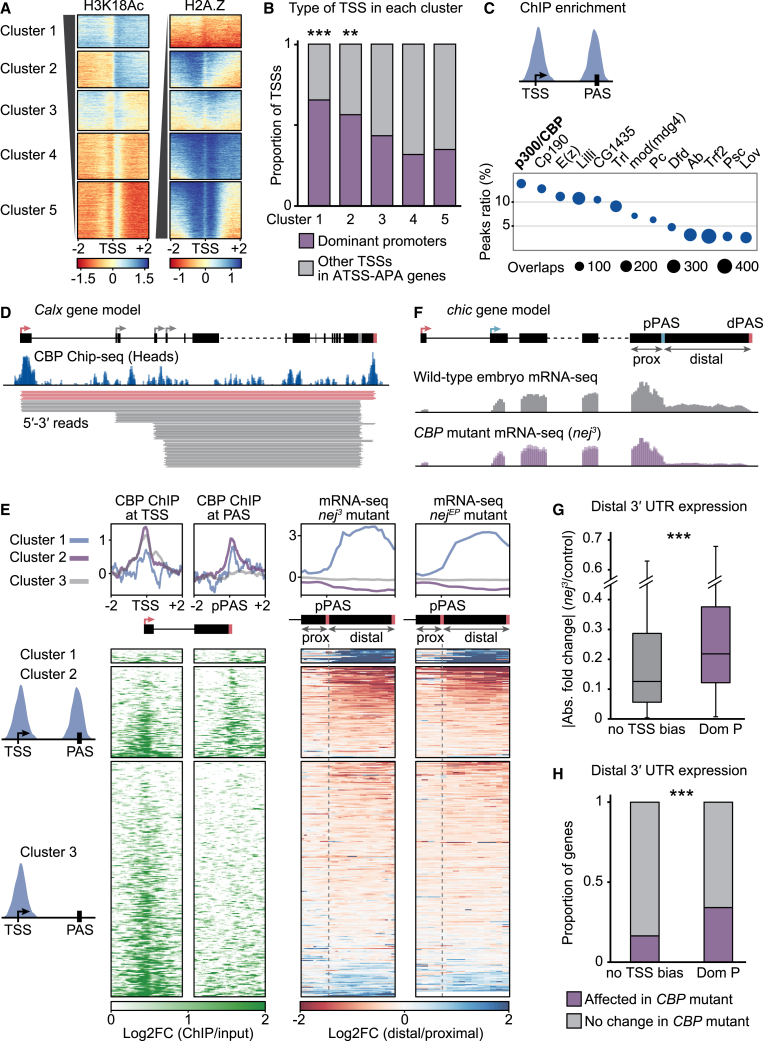
TSSs exert promoter dominance through specific chromatin signatures (A) Heatmaps representing ChIP-seq signal at TSS ± 2 kb genome-wide for the histone variant H2A.Z and the histone mark H3K18Ac. Genes are grouped by k-means clustering, using both H2A.Z and H3K18Ac signal. On average, ATSS-APA genes measure 20.010 kb from TSS to distal PAS. (B) For each cluster, the proportion of dominant promoters (in ATSS-APA genes) is shown. ^∗∗∗^p < 2.2e−16 and ^∗∗^p = 0.006 (two-tailed Fisher’s exact test). (C) ChIP-seq peak enrichment analysis of the TSS and PAS of dominant promoter isoforms. Factors significantly enriched (adj. p value < 0.01) at both TSS ± 150 nt and PAS ± 150 nt are shown, ranked by the ratio of total peaks that map to dominant promoters. (D) CBP ChIP-seq signal and full-length reads representing distinct 5′-3′ isoforms of the gene *Calnexin* (*Calx*). The dominant promoter, its associated PAS, and long reads of the corresponding 5′-3′ isoform are colored in red. CBP ChIP-seq data from *Drosophila* heads are shown as a log_2_ ratio normalized to input. (E) Enrichment of CBP ChIP-seq signal at transcript TSSs (±2 kb) and proximal PASs (±2 kb) and RNA expression of distal 3′ UTRs in CBP mutants (two independent alleles, *nej*^3^ and *nej*^*EP1179*^), compared with control embryos. mRNA-seq heatmaps and profile plots display 0.5 kb upstream of the proximal PAS (prox) and the distal 3′ UTR downstream (distal, scaled region). Genes are grouped into three clusters by k-means clustering, using both CBP ChIP-seq signal at proximal PASs and mRNA-seq signal in *nej*^3^ and *nej*^*EP1179*^ mutants. mRNA-seq was performed on RNA extracted from 14- to 16-h AEL embryos in four biological replicates for each genotype. (F) mRNA-seq signal tracks of the gene *chickadee* (*chic*), whose distal PAS selection depends on p300/CBP. Dominant promoters for each PAS are indicated in the respective color. ChIP-seq data from *Drosophila* heads are from modENCODE.^61^ (G) 3′ end selection change in *CBP* mutant embryos (*nej*^3^), calculated as the change in mRNA expression of the distal transcript regions, compared with control embryos, for PASs linked to a dominant promoter (Dom P) and those with no TSS bias. ^∗∗∗^p = 6.7e−8 (two-tailed Student’s t test). (H) Proportion of ATSS-APA genes with (Dom P) or without (no TSS bias) dominant promoters in which 3′ end selection was significantly affected (p < 0.05, Wald test) in *CBP* mutant embryos (*nej*^3^). ^∗∗∗^p = 3.5e−11 (two-tailed Fisher’s exact test). See also Figure S5 and Table S4.

We grouped TSS regions genome-wide according to H2A.Z and H3K18ac ChIP-seq signal, which generated five clusters of distinct H2A.Z and H3K18 patterns. Cluster 1 and cluster 2 were characterized by H2A.Z depletion concomitant with H3K18Ac enrichment. Strikingly, those two clusters included significantly more dominant promoters than the other three clusters (Figures 5A and 5B; Table S4), suggesting that H2A.Z depletion and H3K18Ac enrichment are common characteristics of dominant promoters. Next, we assessed transcription factor binding at the TSS and linked 3′ end of dominant promoter genes in fly heads, using the ReMap 2022 database.^63^ We found coupled enrichment of 20 factors at both transcription initiation and termination sites of these genes (Figures S5B–S5D); most interestingly, the highly conserved acetyltransferase Nejire (Nej, also known as p300 or CREB-binding protein, CBP) was the factor most frequently found at dominant promoters and at their associated 3′ end (Figures 5C and 5D). Fly and mammalian CBP promote the proper deposition of H3K18Ac,^64^^,^^65^ the histone mark we found enriched around dominant promoters. Together, our data thus indicate that dominant promoters of ATSS-APA genes are characterized by a specific epigenetic landscape, partially established by the presence of CBP.

### p300/CBP mediates dominant-promoter-driven 3′ end site selection

To test whether CBP is instructive for the selection of alternative PASs, we performed mRNA-seq and assessed 3′ end usage in two independent CBP mutants. We used 14- to 16-h embryos, a stage at which maternally deposited CBP was depleted but embryos still showed a normal gross morphology. The absence of zygotic CBP caused a widespread impairment of the embryonic 3′ end landscape: 21% of all expressed APA genes displayed a change in 3′ end site selection, characterized by a significant upregulation or downregulation of RNA expression downstream of the proximal PAS, compared with upstream regions (Figure 5E). Strikingly, affected genes are those that display, in wild-type flies, CBP ChIP signal at both the TSS and the associated PAS (clusters 1 and 2), whereas APA was largely unaffected in genes where CBP signal was only found at the TSS (cluster 3, Figure 5E; Table S4). PAS shifts were more frequent and more pronounced in dominant-promoter genes compared with TSS-unbiased genes (Figures 5F–5H), demonstrating that p300/CBP mediates, at least partially, dominant-promoter-driven 3′ end site selection. In contrast, mutation of one of three other factors we had found enriched at the TSS and PAS of dominant promoter genes—Enhancer of zeste (E(z)), Deformed (Dfd), and Posterior sex combs (Psc)—had little to no effect on PAS usage (Figure S5E). We propose that in addition to CBP, other factors are involved in the promoter-mediated regulation of APA, both globally and on a gene-by-gene basis. Such factors may include chromatin modifiers, AS regulators, and transcription factors.

### TSS influence on isoform choice is a conserved regulatory mechanism

**Figure 6:**
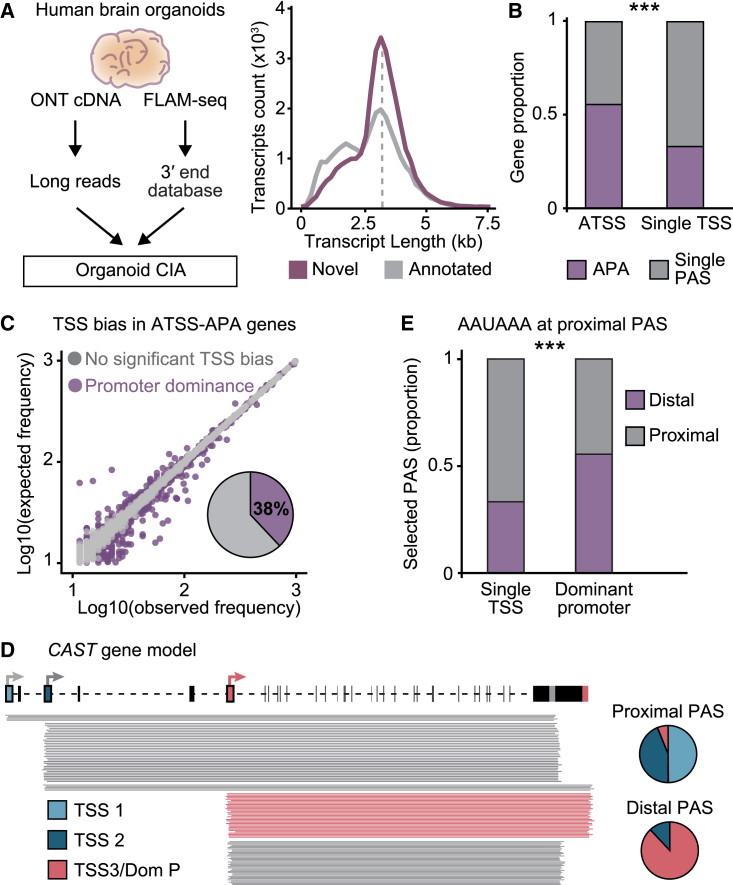
Dominant promoters drive PAS selection in human brain organoids (A) Organoid CIA assembly pipeline. The distribution of novel and previously annotated isoforms as a function of transcript length is indicated (n = 3). (B) Proportion of genes that undergo APA in each TSS category. ^∗∗∗^p < 0.001 (two-tailed Fisher’s exact test). (C) Identification of TSS biases in human brain organoids. The plot was generated as in Figure 3C. 38% of ATSS-APA genes show a significant bias (p < 0.01, chi-squared test with Monte Carlo simulation and Benjamini-Hochberg correction). (D) Representative example of a gene with promoter dominance in brain organoids. Full-length reads represent distinct 5′-3′ isoforms of the gene *CAST*. The dominant promoter, its associated PAS, and long reads of the corresponding 5′-3′ isoform are colored in red in the gene model. Pie charts represent the contributions of each TSS to the expression of each 3′ end. (E) PAS usage when the canonical poly(A) signal AAUAAA is found within a 50-nt window of the most proximal PAS of the gene. Proximal and distal denote PASs found in the proximal 20% or distal 80% of the 3′ UTR, respectively. ^∗∗∗^p < 0.001 (two-tailed Fisher’s exact test). see also Figure S6 and Tables S1–S3 and S4.

**Figure S6:**
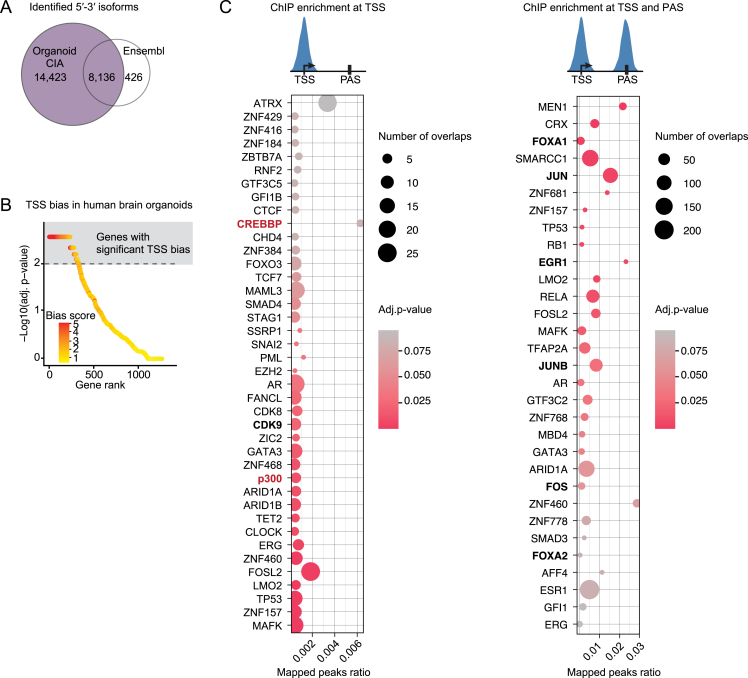
Dominant promoters drive PAS selection in human brain organoids, related to Figure 6 (A) Venn diagram describing the overlap of 5′-3′ isoforms in the Ensembl and CIA reference transcriptomes for human brain organoids (three biological replicates). 5′-3′ isoforms were considered distinct if they differed by more than 50 nt at the 5′ end or 150 nt at the 3′ end. Comparison after gene expression filtering. Organoid CIA identified around 22,000 5′-3′ isoforms. (B) TSS bias in ATSS-APA genes assessed using multinomial testing in human brain organoids. The observed vs. expected counts of 5′-3′ isoforms were used for multinomial testing (chi-squared test with Monte Carlo simulation and Benjamini-Hochberg correction). Genes are represented as dots, ranked by p value and color-coded according to bias score (absolute value of residuals). (C) ChIP-seq peak enrichment analysis at the TSS and PAS of dominant promoter isoforms. Represented are factors significantly enriched (adj. p value < 0.1) at the TSS ± 150 nt (left), and at both the TSS ± 150 and its associated PAS ± 150 nt (right), ranked by the ratio of total peaks that map to the dominant promoter. Transcription factors and co-activators reported to influence 3′ end site choice^12^ are in bold; homologs of p300/CBP are in red. Data are from the ReMap database.^63^

To assess whether TSS-mediated PAS selection is conserved in mammals, we performed our LRS-based analysis in human cerebral organoids, an *in vitro* model of the human brain.^66^ Coupling FLAM-seq with ONT cDNA sequencing and size selection, we generated an organoid CIA dataset including many novel long mRNA isoforms and defined highly accurate 5′-3′ isoforms in ATSS-APA genes (Figures 6A and S6A; Tables S1–S3). Since FLAM-seq identified only 16,840 3′ end sites, we performed 3′ end sequencing (3′-seq) and predicted further confident 3′ end sites based on the nucleotide composition of FLAM 3′ ends, thereby substantially expanding the 3′ end database (see STAR Methods). Similar to *Drosophila*, in human organoids the presence of ATSSs was associated with APA (Figure 6B). We applied LATER to the human dataset and found that over a third of ATSS-APA genes display a TSS bias, in which 3′ end choice is influenced by the promoter (Figures 6C and S6B; Table S4), in many cases mediated by skipping of the proximal canonical poly(A) signal (Figures 6D and 6E). The lack of ChIP-seq data from human neural tissue prevented us from identifying a clear TSS signature of dominant promoters, as we did in *Drosophila*. However, we performed a transcription factor enrichment analysis using the ReMap 2022 database^63^ and found that factors displaying an association with APA,^12^ such as FOXA1 and p300/CBP, were enriched at dominant promoters and/or linked 3′ ends also in human cells (Figure S6C). We conclude that dominant promoters apply a conserved transcriptional constraint on isoform choice, often mediating the usage of more distal PASs. The epigenetic signatures at these sites may have evolved to aid in the recruitment of transcription and processing factors—including p300/CBP—that execute this program, which is determined at the time of transcription initiation.

## Discussion

Over the past decades, a rich body of work has described coupling mechanisms that coordinate transcription with splicing^67^^,^^68^; for example, a role for promoter identity,^69^ RNA Pol II kinetics,^70^ and transcription factors^71^ was demonstrated in defining splice site choice. In comparison, our knowledge on links between transcription initiation and APA was very limited.^19^ In this work, we provide an integrated view of mRNA features and their association in individual transcripts. Our data will serve as a useful resource to study alternative RNA processing, poly(A) tail lengths, RNA modifications, and the interrelation of these features in a tissue-dependent manner. Our finding that 3′ end site selection depends on TSS choice has broad implications for the study of gene expression and its role in disease. It is well established that the use of distinct 3′ end sites contributes to important gene expression programs, including those involved in developmental transitions, tissue identity, and the cell cycle; APA deregulation is associated with numerous human pathologies, most notably cancer.^1^^,^^3^^,^^72^ We hypothesize that the regulation of isoform expression by the use of ATSSs is a central mechanism to ensure tissue function and identity.

Given the pattern of bidirectional 5′-3′ isoform production we found when comparing tissues, it is evident that both *cis*-elements as well as tissue-specific *trans-*factors must act at transcription initiation to drive APA. We describe two modes of APA regulation in *cis*: TSS unbiased, in which the site of transcription termination does not depend on the TSS and is likely determined by *cis*- and *trans*-regulatory elements at the PAS^73^; and promoter dominance, in which the use of specific TSSs drives differential splice site and PAS usage. Coupling 5′ ends with 3′ ends may represent a cellular strategy to ensure the co-occurrence of particular 5′ UTR and 3′ UTR elements in the same mRNA molecule. Post-transcriptional gene regulation including mRNA localization, stabilization, and translation depends not only on the sequence and structural elements found in 5′ and 3′ UTRs^1^^,^^74^ but also on 5′-3′ communication,^75^ either through physical proximity mediated by the concomitant binding of RBPs to both RNA ends (closed-loop model) or through indirect interactions.^76^ Hence, dominant promoters may act to enhance these intramolecular interactions to regulate mRNA expression.

At dominant promoters, H2A.Z depletion, indicative of high transcription rates, frequent chromatin interactions, and lower nucleosome definition^62^ synergizes with the enrichment of the active histone mark H3K18Ac, which was shown to help prime genes for activation during developmental transitions^77^; such increased chromatin accessibility at the TSS and PAS may enhance 5′-3′ coupling and the controlled differential expression of distinct mRNA isoforms. CBP may also link 5′ and 3′ ends independently of its established role in H3K18Ac deposition; concomitant binding of CBP molecules at the TSS and PAS could facilitate an intragenic loop, a mechanism that was proposed to connect transcription initiation with PAS choices.^18^^,^^78^ Additionally, we hypothesize that CBP mediates the recently recognized influence of distal *cis*-regulatory elements on APA,^12^ possibly by binding to enhancer RNAs (eRNAs), an interaction that stimulates histone acetylation and transcription of target genes.^79^ Gene topology may further distinguish the regulation of neuronal ATSS-APA genes. In mouse brains, “melting” chromatin states and distinct chromatin contact patterns were seen in long genes associated with specialized neuronal processes,^80^ and it is possible that such topological constraints contribute to 5′-3′ coupling. We propose that dominant promoters, by residing in a chromatin environment that dictates specialized regulation through enhanced protein interactions and possibly gene looping,^81^ promote communication between the transcription and RNA processing machineries. Interestingly, dominant promoters display typical characteristics of promoters of developmental genes, including lower nucleosome occupancy, CBP binding, and H3K18Ac. “Developmental core promoters” were previously defined as TSSs regulated by “developmental enhancers” that play a defining role in development-, tissue-, or context-specific gene regulation, in contrast with “housekeeping promoters.”^82^ Our results in the context of prior literature are therefore consistent with a model in which developmental genes employ specific epigenetic regulation evolved to ensure robust and highly regulated interactions not only between enhancers and promoters but also between promoters and PASs to dictate gene expression.

Coupling 5′ ends to 3′ ends of transcripts represents a conserved principle in the regulation of gene expression, with broad relevance, as APA affects mRNA coding potential, localization, stability, and translation to achieve context-specific modulation of developmental genes. The universal impact of alternative mRNA processing in the etiology of disease has been highlighted by the substantial association found between APA-altering SNPs in 3′ UTRs with human phenotypic traits and diseases,^83^ which can be further probed using variant expression-aware annotations^84^ and large LRS datasets of human tissues.^27^ Linking 5′ ends to disease-relevant mutations in 3′ UTRs will close an important gap in our understanding of genetic disease mechanisms, aid in the identification of disease-associated mutations in the full-length context in which they are deleterious, and may provide a platform to target variant-associated diseases.

### Limitations of the study

We centered our analyses on the nervous system as a whole, as opposed to considering the complexity of its many different cell types. As a consequence, for genes with extreme isoform diversity and highly cell-type-specific isoform expression, only relatively abundant isoforms passed our stringent detection cutoff. Therefore, we expect that many functionally relevant mRNA isoforms went undetected. Our study uses BluePippin size selection prior to nanopore LRS. Although gene expression calculations from these data were highly consistent with those obtained through mRNA-seq, in individual cases, longer transcripts may be overrepresented. Full-length mRNA coverage from nanopore long reads substantially declined in transcripts exceeding 10 kb in size. Although we excluded isoforms exceeding that limit from quantitative analyses, they are still depicted in the CIA atlas, where they may be underrepresented, compared with significantly shorter mRNAs. Finally, the transcription factor binding analysis on human TSSs conducted with the ReMap 2022 database^63^ used ChIP-seq data from a variety of human cells: the results shown in Figure S6 likely incompletely represent binding in cerebral organoids.

## STAR★Methods

### Key resources table

**Table:** 

REAGENT or RESOURCE	SOURCE	IDENTIFIER
**Critical commercial assays**		

NEBNext® Poly(A) mRNA Magnetic Isolation Module	New England BioLabs	E7490
PCR-cDNA Sequencing Kit	Oxford Nanopore	SQK-PCS109
AMPure XP for PCR Purification	Beckman Coulter	A63881
Dynabeads™ mRNA Purification Kit	Invitrogen	61006
USB poly(A) length assay kit	Thermo Fisher	Cat# 764551KT
RNAClean XP Beads	Beckmann Coulter	Cat# A63987
SMARTScribe Reverse Transcriptase kit	Clontech	Cat# 639537
Advantage 2 DNA polymerase mix	Clontech	Cat# 639201
Direct RNA sequencing kit	Oxford Nanopore	SQK-RNA002
TruSeq® Stranded mRNA Library Prep	Illumina	Cat# 20020595
TruSeq® Stranded Total RNA Library Prep Gold	Illumina	Cat# 20020599
QuantSeq 3′-Seq Library Prep Kit REV	Lexogen	Cat# 016.96

**Deposited data**		

Raw and analyzed LRS and RNA-seq data	This paper	GEO: GSE203583
CIA reference transcriptome data	This paper	GEO: GSE203583
Drosophila reference genome (dm6)	The FlyBase Consortium/Berkeley Drosophila Genome Project/Celera Genomics	https://www.ncbi.nlm.nih.gov/assembly/GCF_000001215.4/
Human reference genome (GRCh38/hg38)	Genome Reference Consortium	https://www.ncbi.nlm.nih.gov/assembly/GCF_000001405.26/
FLAM-seq and mRNA-seq Human Brain Organoids	Rybak-Wolf et al.^85^	GEO: GSE163952
mRNA-seq embryo (14-16 h and 18-22 h)	Carrasco et al.^86^	GEO: GSE146986

**Experimental models: Cell lines**		

Human iPSC lines iPSC-1 XM001	Thermo Fisher Scientific	A18944
Human iPSC lines iPSC-2	Thermo Fisher Scientific	A18945

**Experimental models: Organisms/strains**		

*D. melanogaster*: *w*^*1118*^	Bloomington Drosophila Stock Center	BDSC: 5905; RRID:BDSC_5905
*D. melanogaster*: GFP-marked TM3 balancer: w[1118]; Dr[Mio]/TM3, P{w[+mC]=GAL4-twi.G}2.3, P{UAS-2xEGFP}AH2.3, Sb[1] Ser[1]	Bloomington Drosophila Stock Center	BDSC: 6663; RRID:BDSC_6663
*D. melanogaster*: *orb*^*ΔDP*^	This paper	N/A
*D. melanogaster*: *tub-Gal4;UAS:dCas9-VPR*: w[^∗^]; P{y[+t7.7] w[+mC]=UAS-3xFLAG.dCas9.VPR}attP40; P{w[+mC]=tubP-GAL4}LL7/T(2;3)TSTL14, SM5: TM6B, Tb[1]	Bloomington Drosophila Stock Center	BDSC: 67048; RRID:BDSC_67048
*D. melanogaster*: *Mvl-sgRNA* y[1] sc[^∗^] v[1] sev[21]; P{y[+t7.7] v[+t1.8]=TOE.GS01237}attP40	Bloomington Drosophila Stock Center	BDSC: 78119; RRID:BDSC_78119
*D. melanogaster*: *ttv-sgRNA* y[1] sc[^∗^] v[1] sev[21]; P{y[+t7.7] v[+t1.8]=TOE.GS01385}attP40	Bloomington Drosophila Stock Center	BDSC: 78207; RRID:BDSC_78207
*D. melanogaster*: *ttk-sgRNA* y[1] sc[^∗^] v[1] sev[21]; P{y[+t7.7] v[+t1.8]=TOE.GS02363}attP40	Bloomington Drosophila Stock Center	BDSC: 78287; RRID:BDSC_78287
*D. melanogaster*: Fatp1*-sgRNA* y[1] sc[^∗^] v[1] sev[21]; P{y[+t7.7] v[+t1.8]=TOE.GS01376}attP40	Bloomington Drosophila Stock Center	BDSC:79440; RRID:BDSC_79440
*D. melanogaster*: wun*-sgRNA* y[1] sc[^∗^] v[1] sev[21]; P{y[+t7.7] v[+t1.8]=TOE.GS01590}attP40	Bloomington Drosophila Stock Center	BDSC: 79461; RRID:BDSC_79461
*D. melanogaster*: chn*-sgRNA* y[1] sc[^∗^] v[1] sev[21]; P{y[+t7.7] v[+t1.8]=TOE.GS02080}attP40	Bloomington Drosophila Stock Center	BDSC: 79871; RRID:BDSC_79871
*D. melanogaster*: non-targeting *sgRNA* y[1] sc[^∗^] v[1] sev[21]; P{y[+t7.7] v[+t1.8]=GS00089}attP40	Bloomington Drosophila Stock Center	BDSC: 67539; RRID:BDSC_67539
*D. melanogaster*: csw*-sgRNA* y[1] sc[^∗^] v[1] sev[21]; P{y[+t7.7] v[+t1.8]=TOE.GS01896}attP40	Bloomington Drosophila Stock Center	BDSC: 78649 RRID:BDSC_78649
*D. melanogaster*: zfh1*-sgRNA* y[1] sc[^∗^] v[1] sev[21]; P{y[+t7.7] v[+t1.8]=TOE.GS02033}attP40	Bloomington Drosophila Stock Center	BDSC: 79798 RRID:BDSC_79798
*D. melanogaster*: sbb*-sgRNA* y[1] sc[^∗^] v[1] sev[21]; P{y[+t7.7] v[+t1.8]=TOE.GS02147}attP40	Bloomington Drosophila Stock Center	BDSC: 79903 RRID:BDSC_79903
*D. melanogaster*: twin*-sgRNA* y[1] sc[^∗^] v[1] sev[21]; P{y[+t7.7] v[+t1.8]=TOE.GS02161}attP40	Bloomington Drosophila Stock Center	BDSC: 79908 RRID:BDSC_79908
*D. melanogaster*: jing*-sgRNA* y[1] sc[^∗^] v[1] sev[21]; P{y[+t7.7] v[+t1.8]=TOE.GS02847}attP40	Bloomington Drosophila Stock Center	BDSC: 80271 RRID:BDSC_80271
*D. melanogaster*: psq*-sgRNA* y[1] sc[^∗^] v[1] sev[21]; P{y[+t7.7] v[+t1.8]=TOE.GS05187}attP40	Bloomington Drosophila Stock Center	BDSC: 82755 RRID:BDSC_82755
*D. melanogaster*: CASK*-sgRNA* y[1] sc[^∗^] v[1] sev[21]; P{y[+t7.7] v[+t1.8]=TOE.GS01254}attP40	Bloomington Drosophila Stock Center	BDSC: 78127 RRID:BDSC_78127
*D. melanogaster*: sky*-sgRNA* y[1] sc[^∗^] v[1] sev[21]; P{y[+t7.7] v[+t1.8]=TOE.GS02377}attP40	Bloomington Drosophila Stock Center	BDSC: 78295 RRID:BDSC_78295
*D. melanogaster*: Pka-R1*-sgRNA* y[1] sc[^∗^] v[1] sev[21]; P{y[+t7.7] v[+t1.8]=TOE.GS01286}attP40	Bloomington Drosophila Stock Center	BDSC: 78595 RRID:BDSC_78595
*D. melanogaster*: Pdp1*-sgRNA* y[1] sc[^∗^] v[1] sev[21]; P{y[+t7.7] v[+t1.8]=TOE.GS02089}attP40	Bloomington Drosophila Stock Center	BDSC: 79516 RRID:BDSC_79516
*D. melanogaster*: SPoCk*-sgRNA* y[1] sc[^∗^] v[1] sev[21]; P{y[+t7.7] v[+t1.8]=TOE.GS01261}attP40	Bloomington Drosophila Stock Center	BDSC: 79673 RRID:BDSC_79673
*D. melanogaster*: Mef2*-sgRNA* y[1] sc[^∗^] v[1] sev[21]; P{y[+t7.7] v[+t1.8]=TOE.GS02062}attP40	Bloomington Drosophila Stock Center	BDSC: 79863 RRID:BDSC_79863
*D. melanogaster*: brat*-sgRNA* y[1] sc[^∗^] v[1] sev[21]; P{y[+t7.7] v[+t1.8]=TOE.GS02140}attP40	Bloomington Drosophila Stock Center	BDSC: 79900 RRID:BDSC_79900
*D. melanogaster*: REPTOR*-sgRNA* y[1] sc[^∗^] v[1] sev[21]; P{y[+t7.7] v[+t1.8]=TOE.GS02742}attP40	Bloomington Drosophila Stock Center	BDSC: 79987 RRID:BDSC_79987
*D. melanogaster*: E2f1*-sgRNA* *y[1] sc[^∗^] v[1] sev[21]; P{y[+t7.7] v[+t1.8]=SAM.dCas9.GS02441}attP40*	Bloomington Drosophila Stock Center	BDSC: 80516 RRID:BDSC_ 80516
*D. melanogaster*: Stat92E*-sgRNA* y[1] sc[^∗^] v[1] sev[21]; P{y[+t7.7] v[+t1.8]=SAM.dCas9.GS02442}attP40/CyO	Bloomington Drosophila Stock Center	BDSC: 80517 RRID:BDSC_80517
*D. melanogaster*: gfzf*-sgRNA* y[1] sc[^∗^] v[1] sev[21]; P{y[+t7.7] v[+t1.8]=SAM.dCas9.GS05528}attP40	Bloomington Drosophila Stock Center	BDSC: 84063 RRID:BDSC_84063
*D. melanogaster*: *nej*^3^ mutant w[^∗^] nej[3]/FM7c	Bloomington Drosophila Stock Center	BDSC:3729 RRID:BDSC_3729
*D. melanogaster*: *nej*^*EP1179*^ mutant w[^∗^] P{w[+mC]=EP}nej[EP1179]	Bloomington Drosophila Stock Center	BDSC: 30733; RRID:BDSC_30733
*D. melanogaster*: *E(z)*^*731*^mutant w[^∗^]; E(z)[731] P{1xFRT.G}2A/TM6C, Sb[1] Tb[1]	Bloomington Drosophila Stock Center	BDSC: 24470; RRID:BDSC_24470
*D. melanogaster*: *psc*^h27^ mutant Psc[h27]/CyO	Bloomington Drosophila Stock Center	BDSC: 5547; RRID:BDSC_5547
*D. melanogaster*: *psc*^e22^ mutant Psc[e22]/CyO	Bloomington Drosophila Stock Center	BDSC: 5546; RRID:BDSC_5546
*D. melanogaster*: *Dfd*^1^ mutant Dfd[1] p[p]	Bloomington Drosophila Stock Center	BDSC: 800; RRID:BDSC_800
*D. melanogaster*: *Spps*^*G8810*^mutant w[1118]; P{w[+mC]=EP}Spps[G8810]/TM6C, Sb[1]	Bloomington Drosophila Stock Center	BDSC:30186; RRID:BDSC_30186

**Oligonucleotides**		

Oligonucleotides used for RT-qPCR	Table S6	N/A
CRISPR guide RNAs	STAR Methods	N/A

**Software and algorithms**		

Iso-seq3 pipeline	PacBio	https://github.com/PacificBiosciences/IsoSeq
CIA assembly pipeline	This paper	https://doi.org/10.5281/zenodo.7759448 https://github.com/hilgers-lab /CIAtranscriptome_assembly
Long-reads-based Alternative Termination Estimation and Recognition (LATER)	This paper	https://doi.org/10.5281/zenodo.7759430 https://github.com/hilgers-lab/LATER
Long-reads-based Alternative Splicing Estimation and Recognition (LASER)	This Paper	https://doi.org/10.5281/zenodo.7759428 https://github.com/hilgers-lab/LASER
R 4.1.1	N/A	https://www.R-project.org/
Minimap2 v2.17-r941	Li^87^	https://github.com/lh3/minimap2
NanoPlot 1.29.1	N/A	https://github.com/wdecoster/NanoPlot
guppy-5.0.7 model: dna_r9.4.1_450bps_sup.cfg	Oxford Nanopore	https://github.com/nanoporetech/pyguppyclient
snakePipes v1.2.2	Bhardwaj et al.^88^	https://github.com/maxplanck-ie/snakepipes/blob/develop/docs/index.rst
DEXSeq_1.28.3	Anders et al.^89^	http://bioconductor.org/packages/release/bioc/html/DEXSeq.html
DESeq2	Love et al.^90^	N/A
Seurat V4.1.0	N/A	https://github.com/satijalab/seurat/
STARlong v2.7.8a	Dobin et al.^91^	https://github.com/alexdobin/STAR/blob/master/bin/Linux_x86_64/STARlong
STAR v2.6.1b	Dobin et al.^91^	https://github.com/alexdobin/STAR
FLAMAnalysis	Legnini et al.^38^	https://github.com/rajewsky-lab/FLAMAnalysis
pipeline-polya-ng	Oxford Nanopore	https://github.com/nanoporetech/pipeline-polya-ng
GenomicRanges_1.32.7	Lawrence et al.^92^	https://bioconductor.org/packages/release/bioc/html/GenomicRanges.html
GenomicFeatures_1.36.4	Lawrence et al.^92^	https://bioconductor.org/packages/release/bioc/html/GenomicFeatures.html
ggplot2_3.2.1	N/A	https://github.com/tidyverse/ggplot2
dplyr_1.0.8	N/A	https://github.com/tidyverse/dplyr
seqtk 1.2-r94	N/A	https://github.com/lh3/seqtk
Tama	N/A	https://github.com/GenomeRIK/tama
Sierra	Patrick et al.^93^	https://github.com/VCCRI/Sierra
SUPPA v2.3	Trincado et al.^94^	https://github.com/comprna/SUPPA
BSgenome.Dmelanogaster.UCSC.dm6	N/A	https://bioconductor.org/packages/release/data/annotation/html/BSgenome.Dmelanogaster.UCSC.dm6.html
Rsamtools_2.10.0	N/A	https://bioconductor.org/packages/Rsamtools
samtools 1.12	N/A	https://github.com/samtools/htslib.git
UpSetR 1.4.0.	N/A	http://github.com/hms-dbmi/UpSetR
flair v1.1	Tang et al.^40^	https://github.com/BrooksLabUCSC/flair
Biostrings 2.62.0	N/A	https://bioconductor.org/packages/Biostrings
cellranger-6.1.2	Zheng et al.^95^	N/A
snakemake 7.0.4	N/A	https://github.com/snakemake/snakemake
bedtools v2.27.0	N/A	https://github.com/arq5x/bedtools2
vegan 2.6-2	Oksanen et al.^96^	https://github.com/vegandevs/vegan
ReMapEnrich	N/A	https://github.com/remap-cisreg/ReMapEnrich
SQANTI3 v1.2	Tardaguila et al.^21^	https://github.com/ConesaLab/SQANTI3
SQANTI3 v5.1.3	Tardaguila et al.^21^	https://github.com/ConesaLab/SQANTI3
IsoAnnotLite 2.7.3	N/A	https://isoannot.tappas.org/isoannot-lite/
cDNA_Cupcake v12.5	N/A	https://github.com/Magdoll/cDNA_Cupcake
deeptools 3.5.0	N/A	https://github.com/deeptools/deepTools
randomForest	N/A	https://cran.r-project.org/web/packages/randomForest/index.html
MEME Suite 5.5.0 AME	N/A	https://meme-suite.org/meme/tools/ame
MEME Suite 5.5.0 FIMO	N/A	https://meme-suite.org/meme/tools/fimo
exaR/apa_target_caller	Carrasco et al.^86^	https://github.com/hilgers-lab/apa_target_caller
prody 2.2.0	Zhang et al.^97^	http://prody.csb.pitt.edu/
GenomicScores	Puigdevall and Castelo^98^	https://bioconductor.org/packages/release/bioc/html/GenomicScores.html
ChIPseeker	Wang et al.^99^	https://bioconductor.org/packages/release/bioc/html/ChIPseeker.html
TargetScan Fly v7.2	Agarwal et al.^100^	https://www.targetscan.org/fly_72/
DAVID Knowledgebase v2022q4	N/A	https://david.ncifcrf.gov/tools.jsp
Co-evolution analysis	This paper	https://doi.org/10.5281/zenodo.7759440 https://github.com/hilgers-lab/isoform-coevolution
Random forest classification of 3ʹ ends	This paper	https://doi.org/10.5281/zenodo.7438383
Gsignal	N/A	https://github.com/gjmvanboxtel/gsignal

**Other**		

Drosophila mRNA isoform atlas of CIA Transcriptome	This paper	https://hilgerslab.shinyapps.io/ciaTranscriptome/
Isoform-level functional feature annotation of CIA Transcriptome	This paper	GEO: GSE203583
CIA transcriptome explorer	This paper	https://doi.org/10.5281/zenodo.7759434 https://github.com/hilgers-lab/ciaTailoR

### Resource availability

#### Lead contact

Further information and requests for resources and reagents should be directed to and will be fulfilled by the lead contact, Valérie Hilgers (hilgers@ie-freiburg.mpg.de).

#### Materials availability

All plasmids and fly strains generated in this study are available from the lead contact without restriction.

### Experimental model and subject details

#### Drosophila melanogaster

Experiments in this study used male and female (in equal amounts, except for experiments using ovaries) *Drosophila melanogaster* embryos and adult flies. Flies were raised at 25°C. The CIA reference transcriptome was built using *w*^*1118*^ flies (Bloomington stock number 5905). Flies mutant for *p300/cbp/nej* (*nej*^3^ and *nej*^*EP1179*^),^101^^,^^102^ *Enhancer of zeste* (*E(z)*^731^),^103^ *Posterior sex combs* (*Psc*^*h27*^ and *Psc*^*e22*^),^104^ *Deformed* (*Dfd*^1^)^105^ and *Spps* (*Spps*^*G8810*^)^106^ were obtained from the Bloomington Drosophila Stock Center. We used CRISPR/Cas9-mediated genome editing following the procedure described in Port and Bullock ^107^ to generate the *orb* dominant promoter deletion *orb*^*ΔDP*^. Two guide RNAs (GAGAGAGCTCTACATCAGC, CGCCACGGCGTGCAACGCTG) targeted the *orb* promoter region, generating a 1.9 kb deletion beginning 40 bp upstream of the annotated TSS. All embryo injections were performed by Bestgene, Inc. Recessive lethal mutations were kept in heterozygosis over GFP-balancer alleles. In CRISPRa, to induce the expression of tissue-specific TSSs, TRiP-OE lines from the Transgenic RNAi Project^46^^,^^108^ were used. Flies expressing single guide RNAs (sgRNAs) targeting the upstream TSS of genes of interest (sgRNA, example genotype: y[1] sc[^∗^] v[1] sev[21]; P{y[+t7.7] v[+t1.8]=TOE.GS02080}attP40) were crossed with flies expressing, under control of tubulin-Gal4, a catalytically dead Cas9 (dCas9) fused to the VP64 activation domain (Tub>dCas9-VPR, genotype: w; UAS:dCas9-VPR; tub-Gal4/SM5, TM6B). All fly strains are listed in the key resources table.

#### Human cerebral organoids

iPSC-derived cerebral organoids were generated as described in Giandomenico et al.,^109^ with some modifications. Briefly, after dissociation into a cell suspension with accutase, 6,000 cells were seeded per one well of 96-well plates in 100 μl of embryoid body medium (EBM: DMEM/F12, 20% Knockout replacement serum, 1x Glutamax, 1x MEM-NEAA, 2% ESC FBS, 50μM ROCK Inhibitor, 10 μM bFGF). On day four, the medium was replaced with EBM without bFGF and ROCK inhibitor. On day five, the medium was replaced with a neural induction medium (NIM: DMEM/F12, 1x N2 supplement, 1x Glutamax, 1x MEM-NEAA, 10μg/ml heparin solution). On day 7-9, the formed organoids were embedded into Matrigel (Corning, 356234) and kept in NIM for one day, and in 1:1 NIM: organoid differentiation medium (ODM: 1:1 DMEM/F12: Neurobasal, 1xN2 supplement, 1x B27- vitamin A supplement, insulin, 2-ME solution, Glutamax, MEM-NEAA) for one additional day, followed by four days in ODM. Next, the organoids were transferred to ultra-low attachment 6-well plates and cultured on an orbital shaker (85 rpm) in organoid maturation medium (OMM: 1:1 DMEM/F12: Neurobasal, N2 supplement, B27+ vitamin A supplement, insulin, 2-ME solution, Glutamax supplement, MEM-NEAA, Vitamin C solution, chemically defined lipid concentrate, BDNF, GDNF, cAMP, 1% Matrigel).

### Method details

#### Sample collection for RNA analysis

For head transcriptomes, 3-day-old *w*^*1118*^ flies were collected and flash-frozen in liquid nitrogen and heads were homogenized in QIAzol Lysis Reagent (QIAGEN 79306) for RNA extraction. For ovary transcriptomes, 3-day old *w*^*1118*^ virgin females were collected, and 20 ovaries per replicate were dissected and homogenized. For embryo transcriptomes, eggs from *w*^*1118*^ flies were collected for two hours on agar plates and aged for either 14h (14-16h AEL embryos), or 18h (18-20h AEL) at 25°C. 50 embryos per replicate were homogenized. For *orb*^*ΔDP*^, *nej*^3^, *nej*^*EP1179*^*, E(z)*^*731*^, *Psc*^*h27*^, *Psc*^*e22*^*, Dfd*^1^ and *Spps*^*G8810*^ mutant analysis, eggs from mutant flies grown in heterozygosis with GFP-marked balancer chromosomes were collected for two hours on agar plates and aged for the appropriate amount of time at 25°C. Embryos were dechorionated following standard procedures and placed on a plate containing halocarbon oil. 20 to 30 mutant embryos were hand-sorted according to morphology and against GFP signal, in at least three replicates. For the CRISPRa experiment, to obtain flies ubiquitously expressing dCas9 and a promoter-targeting sgRNA, tub>dCas9VPR virgin female flies were crossed with sgRNA males. Crosses were maintained at 25°C and parents were removed from the vial after two days. Eclosed progeny were aged for five days, selected against Tb and Cyo, and the heads and ovaries of five female flies per replicate were processed for RNA extraction. A sgRNA line targeting a non-Drosophila sequence was used as a control. Organoid RNA was prepared from 60-day-old cerebral organoids as described in Rybak-Wolf et al.^85^ Briefly, organoids were collected in TRIzol (Invitrogen 15596026) and RNA was prepared with the Direct-zol RNA Miniprep kit (Zymo Research R2050) according to the manufacturer’s instructions.

#### RNA extraction and RT-qPCR

For all experiments, RNA was extracted using QIAzol Lysis Reagent (QIAGEN 79306) according to the manufacturer’s instructions. Before library preparation, RNA integrity was analyzed using a 2100 Bioanalyzer (Agilent Technologies). Only RNAs with RQN values of 10 were used for all sequencing experiments. For RT-qPCR, 300 ng total RNA were used for reverse transcription with iScript gDNA Clear cDNA Synthesis Kit (Bio-Rad). RT-qPCR was performed in a LightCycler 480 II instrument using FastStart SYBR Green Master (Roche). RT-qPCR primer sequences are listed in Table S6.

#### Short-read Sequencing (RNA-seq and 3ʹ-seq)

Libraries for mRNA-seq were prepared from 3-day-old *w*^*1118*^ fly heads with 100 ng of total RNA using TruSeq Stranded mRNA Library Prep (Illumina 20020595) according to the manufacturer’s instructions. Libraries for total RNA-seq were prepared from dissected fly ovaries with 100 ng of total RNA using TruSeq Stranded total RNA Library Prep (Gold) (Illumina 20020599) according to the manufacturer’s instructions. Paired-end sequencing was performed using the NovaSeq6000 platform (Illumina) and 101-bp reads. mRNA-seq data from 14-16h AEL embryos and 18-20h AEL embryos are from Carrasco et al.^86^ Sequencing data were processed using the RNA-seq module from snakePipes,^88^ adding flags for --trim, -m “alignment-free,alignment”. Reads were mapped to the *Drosophila melanogaster* reference genome (Ensembl assembly release dm6), and the transcriptome reference annotation release-96 using STAR.^91^ 3ʹ-seq libraries were prepared with 10 ng of total RNA using the QuantSeq 3ʹ-seq Library Prep Kit REV (Lexogen) according to the manufacturer’s instructions. Paired-end sequencing was performed using the NovaSeq6000 platform (Illumina) and 101-bp reads.

#### Nanopore sequencing (ONT cDNA)

Nanopore sequencing was performed on 3-day-old *w*^*1118*^ fly heads, 14-16h AEL embryos, 18-20h AEL embryos, dissected fly ovaries, and human cerebral organoids. For generation of full-length cDNA libraries, polyadenylated RNA molecules were isolated from total RNA preparations using the NEB’s NEBNext® Poly(A) mRNA Magnetic Isolation Module (NEB). Purified polyadenylated RNA molecules were used for library preparation using the cDNA-PCR Sequencing protocol (Oxford Nanopore Technologies). The following modifications were made to the procedure. To eliminate short reads from the final data, both input polyadenylated RNA molecules and cDNA molecules were cleaned upon further processing using AMPure XP beads (Beckman Coulter) using a magnetic bead sample ratio of 0.4. To retain cDNA fragments > 3kb, the BluePippin device and appropriate separation DNA gel cassettes were used (Sage science). cDNA was amplified using 14 PCR cycles and 12 min extension time at 65°C. Libraries were sequenced on a MinION 1B or GridION sequencing device from Oxford Nanopore Technologies (R9.4.1). Reads were processed using guppy-5.0.7 (model: dna_r9.4.1_450bps_sup.cfg). Reads were aligned to the *Drosophila melanogaster* reference genome (Ensembl assembly release dm6) or to the *Homo sapiens* reference (GRCh38), and transcriptome reference annotation release-96 and release-91, respectively. For genomic alignments, reads were mapped using minimap2,^87^ with parameters “minimap2 -ax splice -u f”. Alignment files were sorted and indexed using samtools v1.12. For transcriptome alignments, “minimap2 -ax map-ont -u f” was used.

#### Nanopore Direct RNA sequencing (DRS)

DRS was performed on 3-day-old *w*^*1118*^ fly heads, 14-16h AEL embryos, and dissected ovaries. Polyadenylated RNA molecules were isolated from total RNA preparations using the Dynabeads™ mRNA Purification Kit (Invitrogen). Multiple poly-A+ pulldowns were pooled to reach 500 ng PolyA+ RNA input for library preparation using the Direct RNA sequencing kit (Oxford Nanopore Technologies). Libraries were sequenced on a MinION 1B or GridION sequencing device from Oxford Nanopore Technologies. Reads were processed using guppy-5.0.7 (model: rna_r9.4.1_70bps_hac.cfg).

#### Iso-seq

Iso-seq libraries were prepared using 500 ng total RNA from 3-day-old *w*^*1118*^ fly heads, processed with the Iso-seq express 2.0 workflow (PacBio) with 14 cycles of PCR amplification and size selection with the BluePippin system for transcripts larger than 3 kb according to the manufacturer’s protocol. After SMRTbell adapter addition, libraries were sequenced on three SMRTcells on a Sequel I PacBio sequencer. The raw data files were processed with SMRT Link v8 software to generate CCS fastq files. Data analysis was performed using the Iso-seq3 pipeline to generate consensus reads. Reads were mapped using STARlong^91^ to the *Drosophila melanogaster* reference genome (Ensembl assembly release dm6), and the transcriptome reference annotation release-96.

#### FLAM-seq

FLAM-seq libraries were prepared as described in Legnini et al.^38^ (extended protocol available at *10.21203/rs.2.10045/v1*) using 4 μg total RNA from 3-day-old *w*^*1118*^ fly heads. Briefly, poly(A)-selected RNA was tailed using the USB poly(A) length assay kit (Thermo Fisher), cleaned up with RNAClean XP Beads (Beckmann Coulter) and reverse transcribed with SMARTScribe Reverse Transcriptase kit (Clontech). The resulting cDNA was purified with XP DNA beads (Beckmann Coulter), amplified by PCR with the Advantage 2 DNA polymerase mix (Clontech), and purified again using Ampure XP DNA Beads (Beckmann Coulter). After SMRTbell adapter addition, libraries were sequenced on 3 SMRT cells on a Sequel I PacBio sequencer. Reads were processed using the FLAMAnalysis pipeline^38^ (https://github.com/rajewsky-lab/FLAMAnalysis) with the *Drosophila melanogaster* Ensembl genome assembly and transcriptome reference annotation (release dm6).

#### Comparison across LRS methods

Calculations of transcript coverage per read were obtained by dividing the number of aligned nucleotides by the annotated transcript length.^20^ To compare gene expression estimates across long-read and short-read sequencing methods, a variance stabilizing transformation (VST) was applied using the DESeq2^90^ function vst() on raw gene counts data from the different samples. The transformed data was used to compute a PCA using the DESeq2 function plotPCA() with standard parameters. Enrichments relative to TSS and PAS were computed by comparing the total number of reads mapping to TSS or PAS regions divided by the total number of reads assigned to the whole gene.^38^ Poly(A) signal enrichment was obtained by screening for motifs in a 20-nucleotide window of every PAS. Screening followed a hierarchical order based on known poly(A) signals and their strength, with the following rank: AATAAA, ATTAAA, AATATA, AAGAAA, AATACA, AATAGA, AATGAA, ACTAAA CATAAA, GATAAA, TATAAA, TTTAAA. The positional probabilities per nucleotide were computed by counting the total number of times a given nucleotide was found in a given position per total number of nucleotides observed at a given position.

#### Comparison of long read 5ʹ end pile-ups

For the benchmarking of LRS putative novel TSSs, we used ONT cDNA datasets. The reads were trimmed to their most 5ʹ nucleotide, and peaks were called in windows of 50 nt. Only peaks with more than 30 counts per million were kept for comparison. Peaks were tested for overlaps against the Eukaryotic Promoter Database (EPD) using a window of 50 nt.^39^ Using the ChipSeeker package^99^ and a window of -150 to +150, non-overlapping 5ʹ-pile-ups were annotated to features against the reference annotation (Ensembl assembly release dm6).

#### Generation of the Drosophila Combined Isoform Assembly (CIA) database

##### Transcriptome assemblies

For each tissue and method, all sequencing replicates were merged into a FASTQ file before assembly. Minimap2^87^ was used to map Nanopore long reads with the "-ax splice -uf" option to the Drosophila dm6 genome indexed with the "-x 14" option. STARlong^91^ was used to map Iso-seq and FLAM-seq data using the following parameters^38^: “--outFilterMultimapScoreRange 20 --outFilterScoreMinOverLread --outFilterMatchNminOverLread 0.66--outFilterMismatchNmax 1000 --winAnchorMultimapNmax 200 --seedSearchStartLmax 12 --seedPerReadNmax 100000--seedPerWindowNmax 100 --alignTranscriptsPerReadNmax 100000 --alignTranscriptsPerWindowNmax 10000”. The resulting BAM files were indexed and converted to bed12 files. FLAIR^40^ was then used to correct and collapse isoforms. During the FLAIR *correct* step, splice junction information from the respective RNA-seq datasets (short reads) was used to correct individual transcriptomes. During the FLAIR *collapse* step, the Eukaryotic Promoter Database EPD^39^ was used to retain only reads with a supported TSS at their 5ʹ end, using “--max_ends 5” to allow for multiple 5ʹ-3ʹ end identification. A minimum of three (Nanopore) or two (Iso-seq/FLAM-seq) full-length reads were required for an isoform to be collapsed in the assembly. The resulting isoforms were annotated with SQANTI3 v1.2^21^ to determine novel isoforms and structural categories, using an internal priming window of 50.

##### Generation of a PAS database

Assemblies were filtered for 3ʹ ends that likely originated from internal priming or truncation during library preparation. We used FLAM-seq and DRS data, as both of these methods allow for poly(A) tail detection, to perform poly(A) tail calling. Only reads containing a poly(A) tail were retained, and were trimmed to a single nucleotide preceding the poly(A) tail. Single nucleotide reads were clustered in 20-nt windows; clusters supported by at least two reads were included in the PAS database. The database includes only protein-coding transcripts.

##### 3ʹ end filtering and correction

Individual assemblies from each method were corrected using the PAS database. The following filtering parameters were considered: 1) All isoforms overlapping a 3ʹ end in a window of 100 nt were retained, 2) 3ʹ ends found in the assembly more distal than the 3ʹ ends found in the database were retained only if they were within the reference annotation and contained an AATAAA signal. For 3ʹ end correction: 1) 3ʹ UTR bins were created using the PAS database, starting from the end of the open reading frame, between each consecutive PAS, to the most distal PAS. Isoform 3ʹ ends falling within the last bin of the 3ʹ UTR (between the two distal-most PASs) were corrected to the most distal bin, provided the isoform covered more than 10% of the last bin. Assemblies were merged first by tissue, using TAMA.^110^ Isoform merging was allowed if their difference was less than: 150 nt at the 3ʹ end, 50 nt at the 5ʹ end, and 10 nt at exon boundaries. After generating merged transcriptomes per technique per tissue, we combined transcriptomes per tissue to create the CIA assembly. All steps and pipelines used to create CIA can be found in: https://doi.org/10.5281/zenodo.7759448.

#### Functional annotation of CIA transcriptome

To generate an annotation of the CIA transcriptome at isoform-feature level, we used IsoAnnotLite version 2.7.3 with “-novel flag”, using precomputed files for *Drosophila melanogaster* and the CIA reference. Annotated transcriptome data were deposited at NCBI Gene Expression Omnibus (GEO). To explore and retrieve features from the CIA transcriptome, the R package TaiLoR is available at: https://doi.org/10.5281/zenodo.7759434.

#### Generation of the human cerebral organoid CIA database

Organoid CIA was generated using FLAIR^40^ and steps were identical to Drosophila CIA, with the following modifications. The FANTOM TSS database^111^ was used for FLAIR *collapse*. The organoid 3ʹ end database used organoid FLAM-seq data^85^ obtained from biological replicates of the RNA samples from which ONT cDNA data were generated. Short-read correction used organoid mRNA-seq data^85^ obtained from biological replicates of the RNA samples from which ONT cDNA data were generated. A minimum of three full-length reads were required for an isoform to be collapsed in the assembly. The same parameters were used for database building as for Drosophila CIA, except that clusters supported by at least one read were included in the PAS database. The assembled transcriptomes were assessed for novel isoforms as well as structural categories using SQANTI3v.1.2.^21^

#### Poly(A) tail length estimation

For FLAM-seq datasets, poly(A) tail length estimation was performed using https://github.com/rajewsky-lab/FLAMAnalysis.^38^ For DRS datasets, poly(A) tail length estimations were performed using https://github.com/nanoporetech/pipeline-polya-ng. Lengths were summarized at gene level as the median poly(A) tail length per gene. At isoform level, tails were assigned to transcripts and summarized as median poly(A) tail length per transcript.

#### Saturation analysis

Saturation analysis was performed by pooling all ONT cDNA datasets from all tissues and randomly sampling different fractions from 1% to 100% from the raw read files using seqtkV1.2-r94. Then, the CIA framework was applied to each individual fraction. Results were summarized as a fraction of recovered compared to the full set.

#### 3ʹ end and 5ʹ end diversity calculation

The diversity of 3ʹ ends per gene type was estimated with the Shannon and Simpson indexes using the R package vegan.^96^ To assess the regulatory relationship between TSS and PAS diversity, we computed the number of 3ʹ ends found in genes with increasing numbers of 5ʹ ends, and *vice versa*. The matrices of counts for both calculations were provided as input for both Shannon and Simpson index calculation using the function *diversity()*.

#### 3ʹ UTR length comparisons

Differential expression of 3ʹ ends in heads and ovaries was computed using DEXSeq.^89^ The average length of the bins that were significantly differentially expressed was calculated and summarized per gene for each tissue.

#### Long-reads-based Alternative Termination Estimation and Recognition (LATER)

##### Quantification of 5ʹ-3ʹ isoforms

We counted 5ʹ-3ʹ isoforms using GenomicFeatures.^92^ Each ONT cDNA read was assigned to a TSS in a window of 50 nt and to a PAS in a window of 150 nt. Only the reads that mapped to both features were retained and considered full-length reads. Counts were summarized in 5ʹ-3ʹ isoforms, resulting in counts for each 5ʹ-3ʹ combination. For dominant promoter calculations, transcripts longer than 10 kb were not assessed due to lack of full-length coverage.

##### Calculation of TSS bias in APA-ATSS genes

A joint frequency matrix containing the reads of each 5ʹ-3ʹ isoform was summarized and subjected to multinomial testing with chi-squared test. We used Monte-Carlo simulation processing to obtain reliable estimates for the p-values and then corrected them using the Benjamini-Hochberg method. Only genes with at least two 5ʹ-3ʹ isoforms, each isoform defined by at least two full-length reads, were considered for the analysis. For Drosophila data, a gene was classified as transcriptionally biased with the p-value cutoff: adj. p-value < 0.1. For human brain organoid data, because it was supported by fewer reads, we used a more stringent p-value cutoff: adj. p-value < 0.01.

##### Calculation of TSS bias

Promoter dominance was estimated using two different metrics: TSS contribution and PAS contribution. TSS contribution represents the number of reads of a given 5ʹ-3ʹ isoform, divided by the total number of reads supporting the overall expression of that 3ʹ end. PAS contribution represents the number of reads of a given 5ʹ-3ʹ isoform, divided by the total number of reads supporting the overall expression of that 5ʹ end. A TSS was termed a “dominant promoter” if 1) the gene was classified as transcriptionally biased, 2) the TSS contribution exceeded 20%, and 3) the PAS contribution exceeded 60%. The R package LATER with a description of all processing steps can be found in: https://doi.org/10.5281/zenodo.7759430.

##### Quantification of differential 5ʹ-3ʹ isoform expression

5ʹ-3ʹ isoforms were quantified using the LATER counter and summarized as a counts table per pair. The table was provided to the DEXSeq framework^89^ for differential isoform usage, modeling each 5ʹ-3ʹ isoform as an exon feature within a gene group.^112^ To determine whether the changes in 5ʹ-3ʹ isoform expression originated from the TSS, the PAS, or both, differential gene expression was carried out individually for each TSS and PAS, then assigned to each 5ʹ-3ʹ isoform.

#### Long-reads-based Alternative Splicing Estimation and Recognition (LASER)

LASER quantifies the regulatory links between exons, 5ʹ ends and 3ʹ ends. Given that every read represents a full-length transcript, we assessed all features of each read to quantify the frequency of co-occurrence between features using multinomial testing.

##### Quantification of TSS-exon or 3ʹ-exon associations

Reads were filtered to retain only full-length reads using the same parameters as in LATER. For every read, junctions were corrected using short-read sequencing and the reference annotation. Then for each read, a database was created containing all exon junctions as well as the 5ʹ and 3ʹ ends. Using this read to feature assignment, the total reads carrying the combination of a given 5ʹ end with an exon-junction, or 3ʹ end with a given exon junction were summarized.

##### Calculation of TSS-exon or 3′-exon biases

We created a database of exon junctions that considered only exons that are independent of 5ʹ (alternative 1st exon) or 3ʹ regulation (alternative last exon). Only genes containing more than one splice junction combination were retained. A joint frequency matrix containing the total number of counts per 5ʹ-exon or 3ʹ-exon pair was summarized and subjected to multinomial testing as in LATER. As a measure of bias strength, we summarized every residual of each tested combination using the sum of squares for each gene. To classify splicing events associated with links, we classified alternative splicing events in the CIA transcriptome using SUPPA.^94^ Using this annotation, exon junctions associated with the splicing events were extracted. Only junctions with an absolute residual change > 0.7 were considered biased. The R package LASER with a description of all processing steps can be found in: https://doi.org/10.5281/zenodo.7759428.

#### ChIP-seq data analysis

ChIP-seq data obtained from Drosophila head tissue (modENCODE^61^) was analyzed. Fastq files were mapped and processed with snakePipes^88^ using DNA-mapping and the ChIP-seq workflow, adding flags for “--singleEnd and --fragmentLength 50”. Bigwig signal tracks were generated by computing the log_2_ fold change of each ChIP compared to the respective input. Heatmaps, gene profiles and clustering were generated using deeptools.^113^

#### Analysis of transcription factor enrichment at TSSs

TSSs were generated from the CIA reference transcriptome using a 50 nt window. Enrichment of factors at TSSs was estimated using the ReMap2022 databases for Drosophila and Human and the package ReMapEnrich^63^ with, as background, ATSS-APA genes without a dominant promoter. Enrichment was determined with the cutoff: p-value<0.01.

#### Analysis of single-cell RNA-seq data using the CIA 3ʹ end database

Raw data from the single-cell Drosophila brain transcriptome atlas^49^ were mapped using CellRanger.^95^ To generate the matrix of counts, the CIA 3ʹ end database was provided as an input to the Sierra^93^ function CountPeaks(). Per isoform-cell counts were annotated to cell types and clustering information with PeakSeuratFromTransfer() using metadata from Davie et al.^49^ 3ʹ end expression was then summarized per cell type using the Seurat::AverageExpression() function, and normalized using the Seurat::NormalizeData() function with LogNormalize. 3ʹ ends per cell type were considered expressed if they were represented by at least 0.1 normalized counts.

#### Conservation of 5ʹ UTRs and 3ʹ UTRs

PhasCons scores were retrieved using the GenomicScores^98^ R package for Drosophila using reference phastCons tree model for the 27 species.

#### Co-evolution analysis

We determined gene co-evolution maps at the single nucleotide level using pairwise mutational information between positions derived from 27 species alignment tracks from UCSC, with *Drosophila melanogaster* (dm6) as the reference sequence. For the genes *stai* and *Act5C*, we extracted multiple sequence alignments from -1.5 kb to the 3ʹ end of the gene. The retrieved alignments were filtered using the refineMSA function from the ProDy package, keeping sequences with 60% gaps (parameter: rowocc = 0.4) and an identity level of 98% (parameter seqid=0.98), since the alignments spanned the entire gene, including introns. We used mutual information to estimate the probability that a given nucleotide change would be accompanied by another nucleotide change. We normalized the mutual information using the average product correction method (APC)^56^ and implemented in the ProDy python package.^97^ To perform a global analysis of co-evolution, we selected the top 50 dominant promoter and the bottom 50 (by p-value from the LATER analysis). We computed co-evolution using three regions of interest of each gene to reduce computational time: 1) TSS1 (-1kb), 2) TSS2 (-1kb), 3) the entire 3ʹ UTR sequence. To extract the mutual information between each TSS region and the 3ʹ UTR from the co-evolution matrix, we identified the local maxima of normalized mutual information using the function gsignal::findpeaks(x, MinPeakDistance = 2, MinPeakWidth = 2, MinPeakHeight = 0.2) of the R package gsignal. For every gene, we computed the sum of local maxima of the overlapping regions promoter/3ʹUTR. We classified genes as “co-evolving” when the sum of local maxima was in the top 50th percentile of the distribution of the sum of local maxima in the dataset. The code for all steps from extraction to processing and output is available at https://doi.org/10.5281/zenodo.7759440.

#### Identification of differential poly(A) site usage

We identified differential poly(A) site usage, using the APA target caller^86^ with the parameters “min_distance = 100 padj < 0.05”.

#### Motif enrichment in dominant-promoter-associated 3ʹ UTRs

To predict potentially relevant microRNA binding sites (i.e. with a higher likelihood to exert a functional impact on target mRNAs) in dominant-promoter associated, distal 3ʹ UTRs, we used a subset of 65 microRNAs that were 1. highly conserved (node of origin: Diptera) and 2. well expressed in fly heads (at least 1000 cpm) from MirGeneDB v.2.1,^57^ collapsed them into 52 unique 7mer (2-8) seed sequences and computed the number of occurrences of their reverse complementary sequence in either proximal or distal 3ʹ UTR isoforms for a set of 173 dominant-promoter genes for which the distal 3ʹ UTR was uniquely associated with a dominant promoter. RBP enrichment in dominant-promoter-associated distal 3ʹ UTRs was performed on the distal 3ʹ UTR segments using the BSgenome.Dmelanogaster.UCSC.dm6 reference genome package in R. The FASTA files were submitted to the MEME suite server and the AME program was used to calculate enrichment. For the comparisons, proximal 3ʹ UTR segments were used as control sequences. Motif scanning was performed using FIMO with a cut-off p-value < 0.0001, using the motif matrices from^114^ for RBP enrichment. For microRNA enrichment analysis, motif scanning used the miRbase v22 Single Species microRNA database for *Drosophila melanogaster*.^115^ To further assess the regulatory potential of these 6 microRNAs, we first confirmed that they are expressed in fly heads in MiRGeneDB v.2.1,^57^ and of the only two resulting (poorly) expressed in fly heads, dme-miR-2279-5p and dme-miR-9388-5p, we used TargetScan Fly v7.2^100^ to compile a list of predicted binding sites transcriptome-wide for miR-2279-5p. A Gene Ontology analysis was performed on the resulting gene list (mRNAs not expressed in heads were excluded) using DAVID (v2022q4). We defined microRNA targets as genes with a cumulative weighted context score less than -1. Head-expressed genes were used as the background. GO terms with a p-value less than 0.05 (after Bonferroni false discovery rate (FDR) correction) were considered significant.

#### 3′-seq analysis

Reads were processed with fastp to remove poly(A) stretches and then mapped to the dm6 genome using STAR v2.6.1b with modified parameters ("--sjdbOverhang 74 --limitBAMsortRAM 60000000000 --alignIntronMax 1"). In order to eliminate the signal that may come from internal priming, any poly(A) sites overlapping with a strand-specific blacklist region that contained genomic positions with more than 70% As in a 10-bp upstream window were discarded. Regions with high A density within 250 bp of annotated transcription end sites were not included in the blacklist. The remaining single base pair poly(A) sites from all samples with a minimum coverage of 5 reads per sample were grouped, with sites within 15 bp merged into a single poly(A) cluster.

#### Random forest classification of 3ʹ ends

Using 3ʹ-seq clusters, we extracted features from 3ʹ ends identified by FLAM-seq in human organoids. These features included: poly(A) signals at 20 nucleotides upstream from the identified 3ʹ end, the nucleotide content and annotated feature (e.g. 3ʹ UTR, 5ʹ UTR) of the 3ʹ end. We used these features to train a Random Forest model in R using the randomForest package. We created a training set based on FLAM-seq 3ʹ end clusters as our TRUE set and non-overlapping 3ʹ ends as the FALSE set. The model was trained using 1000 trees with 12 random variables set at each split (randomForest(ntree=1000, mtry=12)). The TRUE clusters obtained from classification were then used as a poly(A) database to correct human organoid assemblies. Pretrained models are available at: https://doi.org/10.5281/zenodo.7438383.

### Quantification and statistical analysis

Statistical parameters and tests are reported in the respective figure legends; software used is described in the STAR Methods section and in the key resources table.

### Additional resources

A Drosophila mRNA isoform atlas, depicting all CIA transcript isoforms identified and representing their differential expression in several tissues and developmental stages, is publicly available. https://hilgerslab.shinyapps.io/ciaTranscriptome.

## Data Availability

• All LRS and RNA-seq data have been deposited at NCBI Gene Expression Omnibus (GEO) and are publicly available as of the date of publication. Accession numbers are listed in the key resources table. • All original code has been deposited at Zenodo and is publicly available. DOIs and GitHub links are listed in the key resources table. • Any additional information required to reanalyze the data reported in this paper is available from the lead contact upon request.

## Supplemental information

Document S1. Tables S1–S3 and S5 and supplemental references

Table S4. Summary of 5′–3′ isoforms enriched in heads and ovaries, summary of genes with TSS-PAS, TSS-exon and exon-PAS links, and list of genes represented in ChIP-seq analyses, related to Figures 2, 3, 5 and 6

Table S6. RT-qPCR primers used in this study, related to STAR Methods
