# Supplementary material for: Sites of transcription initiation drive mRNA isoform selection
Source: Cell. 2023 May 25;186(11):2438–2455.e22. doi: 10.1016/j.cell.2023.04.012 (PMC10228280; doi:10.1016/j.cell.2023.04.012)
Supplement: Document S1. Tables S1–S3 and S5 and supplemental references [file mmc1.pdf]

Cell, Volume *186*

Supplemental information

Sites of transcription initiation drive mRNA isoform selection

Carlos Alfonso-Gonzalez, Ivano Legnini, Sarah Holec, Laura Arrigoni, Hasan Can Ozbulut, Fernando Mateos, David Koppstein, Agnieszka Rybak-Wolf, Ulrike Bönisch, Nikolaus Rajewsky, and Valérie Hilgers

Supplemental Tables

Table S1. Summary of the total number of reads collected per tissue and LRS method. Related to Figures 1 and 6.

Organism	Drosophila				Human
Tissue	Heads	Embryos 14-16h	Embryos 18-20h	Ovaries	Brain organoids
**Nanopore (ONT cDNA)**					
Standard	828,444				
BluePippin	39,614,269	5,091,510	3,713,022	4,366,849	5,287,083
**Direct RNA (ONT DRS)**					
Standard	1,028,763	5,111,208		4,623,486	
**FLAM-seq**					
Standard	941,167				1,046,039*
**Iso-seq**					
Standard	20,807				
BluePippin	13,837				

* Data from (Rybak-Wolf et al., 2021).

Table S2. Number of isoforms recovered from ONT cDNA and other long-read sequencing methods in each step of the CIA assembly. Related to Figures 1 and 6.

Method and tissue	Number of isoforms found	Number of isoforms also found with other methods	Number of unique isoforms
**Embryos 14-16h ONT cDNA**	13,326	10,516	2,810
**Embryos 14-16h ONT DRS**	3,165	2,942	223
**Embryos 18-20h ONT cDNA**	11,930	9,602	2,328
**Heads ONT cDNA**	48,115	15,475	32,640
**Heads FLAM-seq**	4,845	4,344	501
**Heads Iso-seq**	3,940	3,608	332
**Heads ONT DRS**	1,090	1,016	74

**Ovaries ONT cDNA**	10,187	8,204	1,983
**Ovaries ONT DRS**	4,043	3,628	415
**Human brain organoids ONT cDNA**	58,059	n/a	n/a

Table S3. Number of genes and distinct transcripts (isoforms) recovered from ONT cDNA data in each step of the CIA assembly. Related to Figures 1 and 6.

**Filtering steps. N genes (N isoforms)**			
**Tissue**	**Assembly without correction**	**Assembly after 5' filtering**	**Assembly after 5' filtering and 3' filtering**
**Heads**	26,807 (138,425)	15,873 (74,482)	9,010 (51,613)
**Embryos 14-16h**	14,868 (36,107)	8,497 (20,701)	6,775 (15,027)
**Embryos 18-20h**	12,896 (27,191)	8,877 (19,262)	6,261 (12,356)
**Ovaries**	9,225 (19,890)	7,255 (13,398)	6,351 (11,376)
**Organoids**	44,786 (126,135)	19,782 (85,855)	12,470 (58,059)

Table S5. Drosophila CRISPRa lines tested. Related to Figure 2.
List of 23 used TRiP-OE lines (Ewen-Campen et al., 2017; Zirin et al., 2020) and result of TSS induction. Each sgRNA line targets the upstream TSS of the indicated genes of interest. sgRNA lines were crossed with flies expressing dCas9-VPR under control of Tubulin-Gal4 (Tub>dCas9-VPR) to induce expression from TSSs linked to a specific 3' end. Of twelve crosses designed to induce nervous-system enriched TSSs, induction was achieved for six (successful), induction was not achieved for five (failed), and one was lethal. Of eleven crosses designed to induce ovary-enriched TSSs, induction was not achieved for either, and three were lethal.

**#**	**BDSC number**	**Targeted gene**	**sequence sgRNA1***	**sequence sgRNA2***	**Phenotype**	**Induction status**
**Nervous-system enriched TSS**						
1	78119	*Mvl*	AAATCTTAGGCG GTATTTCTGGG	ACATTTCTAACGG TAACCTGCGG	linked 3′ end induced	successful
2	78207	*ttv*	GGGAAAGCGAAT ATGGTGGAAGG	GAAGATTGGAATG CATCGCTGGG	linked 3′ end induced	successful
3	78287	*ttk*	CAAGAATCGAAC ATTTGAATGGG	CAAGAATCGAACA TTTGAATGGG	linked 3′ end induced	successful
4	78649	*csw*	GCACAGAATGCT CAGAATGATGG	CACAACGTTTCGA AAATAGCGGG	no	failed

5	79440	*Fatp1*	GCACAGAATGCT CAGAATGATGG	CACAACGTTTCGA AAATAGCGGG	linked 3′ end induced	successful
6	79461	*wun*	ACTGTTTACAGTT ATGGACTTGG	AACAGTCTGCAAA TTGTAGGAGG	linked 3′ end induced	successful
7	79798	*zfh1*	GCCCGAATCACC TCGTACATCGG	ACGGATTCGGTAG AGGACGTCGG	no	failed
8	79871	*chn*	ACGAAATATGGT AAAAACTAAGG	TGCTTAATTTAGG AGCTCAATGG	linked 3′ end induced	successful
9	79903	*sbb*	ACATACATAACTG CGCAATATGG	GAGCGGCATTTCT TTAATGGCGG	no	failed
10	79908	*twin*	TTAAAAAAAGGTT AATATGTCGG	TTAAGGGCATTCG CGGCTAATGG	no	failed
11	80271	*jing*	ACTTGCCACCGC GGACACATCGG	CCCCACGCGACC CTCTCTCTTGG	lethal	n/a
12	82755	*psq*	TTTGCTACGGAC TTTGATATGGG	TTTAATCCTGGAA GCACTTATGG	no	failed
**Ovary-enriched TSS**						
13	78127	*CASK*	CTTAGATACCAA CGGGTTTCTGG	CGGAACTTGGAGT CGGTTCACGG	no	failed
14	78295	*sky*	ACACTCTCCCAC AAAACTGCCGG	CAGGAAAACCGAA CGAACGTTGG	no	failed
15	78595	*Pka-R1*	GGTAAATAGTCC GAAAAAGAGGG	AGATAAAAGCAGT AGCAATGTGG	no	failed
16	79516	*Pdp1*	TTAGTCAGAGTC GTTCGTAGTGG	CTGCGACTCTCCG TTACATAAGG	no	failed
17	79673	*SPoCk*	GTGTGGCCACGA TTGGGCCGAGG	ATATTGGCAACGT GTGATCACGG	no	failed
18	79863	*Mef2*	CTTTAATTTAAAA AATTTCCAGG	TTCTACAACCATA AGTACATTGG	no	failed
19	79900	*brat*	GTCAATGTTGGT ATGACACTTGG	TCGATTTAAGTAG CTAAGGACGG	no	failed
20	79987	*REPTOR*	CAGGTGGCACGT CGATTTGGCGG	GGAAAATAAAGAA TGTAAATAGG	no	failed
21	80516	*E2f1*	CGTGAGCCGATG TTATTTAGTGG	n/a	lethal	n/a
22	80517	*Stat92E*	ATCTAACAATTAG AGCATGTCGG	n/a	lethal	n/a
23	84063	*gfzf*	GTTTAGACGCCG TACGCGTAGGG	n/a	lethal	n/a

* sgRNA1 and sgRNA2 sequences from the TRiP-OE-VPR collection available on Flybase (http://flybase.org/reports/FBlc0003381.html). Where only one sgRNA sequence is mentioned, the line used is from an improved version, the TRiP-OE-flySAM collection, a set of transgenic constructs that each consist of a single sgRNA with MS2 loops (http://flybase.org/reports/FBlc0003482.html).

References

Rybak-Wolf, A., Wyler, E., Legnini, I., Loewa, A., Glažar, P., Kim, S.J., Pentimalli, T.M., Martinez, A.O., Beyersdorf, B., Woehler, A., et al. (2021). Neurodegeneration in human brain organoids infected with herpes simplex virus type 1. bioRxiv. 10.1101/2021.03.05.434122.

Ewen-Campen, B., Yang-Zhou, D., Fernandes, V.R., González, D.P., Liu, L.P., Tao, R., Ren, X., Sun, J., Hu, Y., Zirin, J., et al. (2017). Optimized strategy for in vivo Cas9-activation in Drosophila. Proc Natl Acad Sci U S A 114, 9409-9414. 10.1073/pnas.1707635114.

Zirin, J., Hu, Y., Liu, L., Yang-Zhou, D., Colbeth, R., Yan, D., Ewen-Campen, B., Tao, R., Vogt, E., VanNest, S., et al. (2020). Large-Scale Transgenic Drosophila Resource Collections for Loss- and Gain-of-Function Studies. Genetics 214, 755-767. 10.1534/genetics.119.302964.
